# Making Multimethod Latent State–Trait Models for Random and Fixed Situations Accessible: A Tutorial

**DOI:** 10.1111/jopy.13031

**Published:** 2025-06-16

**Authors:** Dora L. Tinhof, Axel Mayer

**Affiliations:** ^1^ Department of Psychological Methods and Evaluation, Faculty of Psychology and Sport Science Bielefeld University Bielefeld Germany

**Keywords:** latent state–trait theory, multimethod, SEM, shiny app, situations, tutorial

## Abstract

**Objective:**

As more researchers employ longitudinal research designs, which integrate multiple methods and multiple (fixed) situations, the need for appropriate analytical methods arises.

**Method:**

Multimethod latent state–trait models for random and fixed situations (MM‐LST‐RF; Hintz et al. 2019) provide a means with which person characteristics, (fixed) situation, and method effects, as well as their interactions can be studied. While these models are very versatile, their complexity poses a significant hurdle to their implementation.

**Results:**

This tutorial helps facilitate the application of MM‐LST‐RF models. First, we present two simpler methodological approaches in which the full MM‐LST‐RF model is broken down into its (a) multimethod and (b) random and fixed situation components. Key parameters and model coefficients are highlighted using a motivational example. Second, we present a user‐friendly shiny app based on a newly developed R function. Users are walked through the process of specifying, estimating, and interpreting an MM‐LST‐RF model guided by detailed explanations of all specification options and practical use recommendations.

**Conclusion:**

The shiny app facilitates the analysis of data from longitudinal study designs implementing multiple methods and (fixed) situations, helping researchers gain a deeper understanding of psychological constructs.

## 1 Introduction

The interactional perspective has become central to personality psychology. It focuses on interactions between individuals and their environment (Endler and Magnusson 1976; Magnusson and Endler 1977) and addresses core questions of the person–situation debate (Epstein 1983; Kenrick and Funder 1988). Given broad consensus that both person and situation factors are essential to understanding states, traits, and behavior (Bader et al. 2024; Fleeson and Noftle 2008, 2009; Funder 2008; Hertzog and Nesselroade 1987; Mischel and Shoda 1995; Roberts 2009), theoretical and methodological approaches must be developed to integrate these factors and their interactions. However, this perspective may be too narrow in scope. Ozer's (1986) simplified version of Cattell's (1966) data box organizes personality data along four dimensions: persons, situations, responses (behaviors or measures), and time. Ozer's framework highlights the dynamic, context‐dependent nature of personality and the need to assess more than just person and situation factors. In line with this framework, researchers have advocated for longitudinal, multi‐situational, and multimethod research designs that go beyond static, single‐occasion assessments (Eid et al. 2006; Rauthmann et al. 2015; Roberts, Harms, et al. 2006).

Methodological approaches applied to such study designs must be able to formalize the complex relationships across these dimensions. To do so, they need to address four key issues:

1. *Measurement error*: Traditional models using observed scores (e.g., ANOVA, regression) fail to account for measurement error, which can obscure effects and limit detection of potential interactions (Cole and Preacher 2014; Geiser et al. 2015). Latent variable models can help address this issue.
2. *Longitudinal change*: Traits have demonstrated both stability and meaningful change across the lifespan (e.g., Caspi et al. 2005; Roberts, Walton, and Viechtbauer 2006), requiring methods that are able to capture consistency as well as change over time.
3. *Situational influences*: Even stable constructs like the Big Five can vary across situations (e.g., Deinzer et al. 1995; Kane 2013; van Bavel et al. 2016). Adequately capturing such context‐dependent variability requires measurements across multiple situations.
4. *Measurement methods*: Different assessment methods can introduce systematic variance unrelated to the underlying construct (e.g., Chang et al. 2012; DiStefano and Motl 2006, 2009; Hoyt 2000); using a single method may lead to method‐dependent results. Multimethod designs are needed to detect and control for such method effects.

In the following sections, we outline how all four dimensions of the data box can be integrated into a single model that addresses the key issues described above.

### 1.1 Latent State–Trait Theory

Latent state–trait (LST) theory (Steyer et al. 1999, 2015) is a latent variable modeling approach, rooted in classical test theory (Lord and Novick 1968), which assumes that each observed score comprises a systematic “true” component and unsystematic error. LST theory extends this by positing that every measurement occurs in a specific situation, aligning with Ozer's (1986) emphasis on the contextual nature of assessments. To address this, LST models use longitudinal designs with repeated measurements, which allow for a decomposition of “true” scores into two latent components: trait factors—which are stable across occasions—and state residuals—which capture occasion‐specific deviations from the trait. LST models thus separate long‐term stable variance from occasion‐specific variance, accounting for measurement error.

### 1.2 Random vs. Fixed Situations

In LST theory, situations do not need to be selected based on predefined attributes, nor is it necessary to record situational information. Such “unknown” situations can be conceptualized as *random situations* (Geiser et al. 2015) and are captured by the state residuals. Since the characteristics of random situations are neither known to nor controlled by the researcher, state residuals reflect both situation main effects as well as person‐by‐situation interactions (Geiser et al. 2015, 168–169). When measurement occasions are treated as random situations, state residuals additionally confound temporal and situational effects, unless situations are truly random (i.e., not systematically different) rather than merely unknown or unrecorded. When simply aiming to distinguish between trait and occasion‐specific variance, it is sufficient to use study designs, which implement only random situations.

However, to separate main effects of situations from person‐by‐situation interactions, *fixed situations* are needed (Geiser et al. 2015). These situations are predefined or selected based on known attributes, are non‐interchangeable, and are comparable across individuals. Measuring individuals in the same fixed situations on multiple occasions allows for a distinction between systematic situational and temporal effects, while implementing multiple fixed situations separates person‐by‐fixed situation interaction effects from the fixed situation main effects. This enables the analysis of personality across the situational dimension of the data box.

Guidance for selecting meaningful fixed situations can be found in literature on situation taxonomies and person‐by‐situation interactions (Frederiksen 1972; Funder 2008; Rauthmann et al. 2015). In practice, fixed situations can be incorporated in various ways, such as experimental manipulation of situational factors, assessment in naturally occurring situations (e.g., work vs. home), recording of situational attributes with methods like experience sampling (Shiffman et al. 2008), or even using a frame‐of‐reference approach to contextualize the employed measurement instruments (e.g., Bing et al. 2004; Schmit et al. 1995).

### 1.3 Multimethod Assessment

Beyond person and situation factors, the response dimension of the data box emphasizes that the way a construct is measured matters. Different measurement methods can introduce method‐specific variance that is neither attributable to the trait nor the fixed situation. Empirical studies have often found surprisingly low convergence across methods, indicating the presence of method effects (e.g., Meyer et al. 2001; Roberts, Harms, et al. 2006). For example, a person's self‐ratings may differ systematically from how others rate that same person, or responses might shift depending on whether items are positively or negatively framed (e.g., Chang et al. 2012; DiStefano and Motl 2006, 2009; Hoyt 2000). This implies that results from studies using a single method may strongly depend on the chosen measurement method. As a consequence, many researchers have advocated for multimethod designs to improve the validity and generalizability of findings (e.g., Eid et al. 2006, 2009, 2024; Roberts, Harms, et al. 2006; Vazire 2006). Multimethod designs help address the response dimension of the data box by explicitly modeling method factors rather than treating method variance as unexplained error.

### 1.4 An Integrated Framework

Recently, Hintz et al. (2019) introduced a multimethod latent state–trait model for random and fixed situations (MM‐LST‐RF) which combines each of the elements discussed above. The full MM‐LST‐RF model allows for the analysis of person, method, and fixed situation effects as well as their interactions within one model. This makes it a powerful tool for modeling the full structure of the four‐dimensional data box in a single framework and for finding answers to core questions in personality research. However, despite its potential, the MM‐LST‐RF model has seen very limited use in empirical personality research to date. Barriers include the logistical challenges of longitudinal, multi‐situational, multimethod studies as well as the statistical complexity of specifying and interpreting MM‐LST‐RF models. Although the logistical demands of data collection are inherent, the analytic complexity can be reduced through accessible tools and clear guidance.

To help address this complexity and facilitate understanding, we provide a modular introduction to MM‐LST‐RF models. First, we explain their structure and key model components via two simpler LST model extensions: (a) LST models for random and fixed situations (LST‐RF; Geiser et al. 2015) and (b) multimethod LST models (MM‐LST; Courvoisier et al. 2008). Second, we present a user‐friendly shiny web app (Chang et al. 2024), based on a new R function (R Core Team 2024), allowing users to specify, estimate, and interpret MM‐LST‐RF models without advanced statistical programming skills. Guided by a motivational example, we provide a step‐by‐step guide on the specification of MM‐LST‐RF models using the shiny app, with practical advice for implementation and interpretation.

### 1.5 Motivational Empirical Example

We draw on a preregistered longitudinal study^1^ of the Big Five personality traits across offline (real world) and online (digital) contexts to illustrate the MM‐LST‐RF model. Following approval by the Bielefeld University Ethics Committee (Ref. 2023–032), the German version (Danner et al. 2019) of the Big Five Inventory‐2 (BFI‐2; Soto and John 2017b) was used to assess traits on two occasions, about 11 days apart. Each participant completed two context‐specific questionnaires framed as either “in everyday life (offline)” or “on the internet (online).” The order was randomized to control for sequence effects. Participants were asked to recruit peers to provide other‐ratings for both contexts. The final sample included 425 participants who ranged in age from 18 to 61 years (*M* = 24.14, *SD* = 7.27), the majority of whom were women (62.53%) and of German nationality (90%). Most participants had at least a high school diploma (91.56%), and around two‐thirds were students (67.25%).

For our motivational example, we focus on six self‐rated items^2^ (summarized in Table 1) constituting the Negative Emotionality scale in the short version of the BFI‐2 (BFI‐2‐S; Soto and John 2017a). This scale aligns with the Neuroticism trait and captures tendencies toward negative emotions like stress, sadness, or insecurity (John et al. 2008; Soto and John 2017b). It can be further divided into three facets—Anxiety, Depression, and Volatility—each of which is assessed using an equal number of true‐ and false‐keyed items. In our example, Negative Emotionality was measured in two fixed situations (offline & online context), on two occasions, using two methods (true‐ & false‐keyed items), with three indicators each (one for each facet), yielding a total of 24 variables.

**TABLE 1:** Items of the short Big Five Inventory‐2 Negative Emotionality scale.

Position^a^	Item	Facet	Key	Name^b^	Notation^c^
4	Worries a lot	Anxiety	True	NAt	*Y*_*11ts*_
9	Tends to feel depressed, blue	Depression	True	NDt	*Y*_*21ts*_
29	Is temperamental, gets emotional easily	Volatility	True	NVt	*Y*_*31ts*_
19	Is relaxed, handles stress well	Anxiety	False	NAf	*Y*_*12ts*_
24	Feels secure, comfortable with self	Depression	False	NDf	*Y*_*22ts*_
14	Is emotionally stable, not easily upset	Volatility	False	NVf	*Y*_*32ts*_

^a^ Refers to the position of the item in the short Big Five Inventory‐2 questionnaire.

^b^ Each item in the dataset was assessed for both the real and digital world (offline/online) across two measurement occasions, resulting in a total of 24 variables. Prefixes are used to distinguish between the four versions of each variable: “*Of1*,” “*Of2*,” “*On1*,” and “*On2*.” “*Of*” and “*On*” indicate the offline and online contexts, respectively, and the numeral denotes the measurement occasion.

^c^ Model notation for manifest variables *Y*_*imts*_. *i* = indicator, *m* = method, *t* = occasion, *s* = fixed situation.

This empirical example helps us illustrate key features of the MM‐LST‐RF model based on three guiding research questions.

***Research Question 1***: *Do trait levels of Negative Emotionality differ between the online (digital) and offline (real world) context*? The growing integration of digital platforms into professional, educational, and personal life—accelerated by the COVID‐19 pandemic—has made the digital world a central part of daily experience. Compared to the real world, the digital world differs in social norms, anonymity, and self‐presentation due to its unique features such as curated content, customizable profiles, and asynchronous communication (Bayer et al. 2020; McFarland and Ployhart 2015). Recent studies suggest that these differences lead to varying trait levels of the Big Five across contexts, with individuals typically scoring lower online (Blumer and Döring 2012; Bunker and Kwan 2021; Taber and Whittaker 2018, 2020). Our first research question is whether Negative Emotionality scores are lower in the digital world compared to the real world.

***Research Question 2***: *Do trait‐level differences between contexts depend on offline (real world) trait levels*? Previous work suggests that reported differences in personality between online and offline contexts may be moderated by the participant's trait levels. For instance, Blumer and Döring (2012) observed the largest discrepancies among individuals with high offline personality scores. Similarly, Bunker and Kwan (2021) found that high scorers often reported lower levels online, whereas low scorers sometimes reported elevated online traits. These findings suggest that there may be person‐by‐fixed situation interaction effects. Our second research question explores whether such person‐by‐fixed situation interactions exist for Negative Emotionality.

***Research Question 3***: *Does Negative Emotionality generalize across contexts when accounting for method variance*? Although we expect trait‐level differences between the real and the digital world, the correlation of Negative Emotionality across contexts should still be substantial. However, prior research has documented method effects for true‐ and false‐keyed items (e.g., DiStefano and Motl 2006, 2009; Horan et al. 2003); it is unclear whether a high correlation reflects true trait consistency or methodological artifacts (e.g., Kenrick and Funder 1988). Our third research question disentangles method effects from true consistency and assesses the degree to which Negative Emotionality generalizes across online and offline contexts while accounting for method variance.

Having described our motivational example and our guiding research questions, we next turn to the structure and estimation of MM‐LST‐RF models.

### 1.6 The MM‐LST‐RF Model

MM‐LST‐RF models are suitable for study designs where a construct is measured in multiple fixed situations on at least two occasions, using two or more distinct methods, with at least two indicators per method. However, they require specific conditions for appropriate use. We briefly outline these conditions based on recommendations by Hintz et al. (2019, 16–17) and a simulation study evaluating the performance of MM‐LST‐RF models (Tinhof and Mayer 2025, in preparation).

MM‐LST‐RF models can be estimated using continuous and ordinal indicators. For ordinal data, robust estimators like mean‐ and variance‐adjusted weighted least squares are recommended (Beauducel and Herzberg 2006; Flora and Curran 2004), and sparse categories may need to be collapsed (Liu et al. 2017). Sample sizes of 300–500 are generally needed, with larger models requiring more participants. Additional measurement occasions can help mitigate problems caused by smaller samples. Due to the use of multiple measurements taken in different fixed situations across multiple occasions, MM‐LST‐RF models are prone to missing data. Full information maximum likelihood (FIML; Dong and Peng 2013) provides unbiased estimates if data are missing at random. However, if missingness is systematic, FIML may be biased (e.g., Enders 2022; Little and Rubin 2019).^3^ Study designs should thus aim to minimize attrition and include relevant auxiliary variables.^4^

MM‐LST‐RF models are best suited for constructs that show both trait‐ and state‐like variability and involve nonzero method effects. For highly stable constructs or those measured with strongly convergent methods, simpler models may suffice, and redundant factors can be dropped to reduce complexity. The model also assumes non‐interchangeable methods (e.g., self‐ vs. other‐ratings), making it unsuitable for interchangeable methods (e.g., multiple peers, multiple co‐workers); in those cases, alternative models are required (Eid et al. 2009, 2024; Koch et al. 2017). Additionally, although situational attributes can be described on a continuum (Rauthmann et al. 2015), the MM‐LST‐RF model requires fixed situations to be treated as discrete categories. Finally, because the model has not yet been extended to multitrait designs, different constructs must currently be analyzed separately.

Having outlined appropriate domains of application, we now turn to the model's methodological foundations. Table 2 provides an overview and definitions of the manifest and latent variables used. In MM‐LST‐RF models, all observed variables *Y*_*imts*_ (*i* = indicator, *m* = method, *t* = occasion, *s* = fixed situation) are defined as functions of latent trait factors *T*_*11s*_, occasion factors *O*_*11ts*_, and error components *ϵ*_*imts*_. Some observed variables additionally load on trait‐method factors *TM*_*ims*_ and occasion‐method factors *OM*_*mts*_ (Equation 1).

(1) $$\begin{array}{c} Y_{\text{imts}}=\left\{\begin{array}{c} \;T_{11s}+O_{11\mathrm{ts}}+\varepsilon_{11\mathrm{ts}}\;\text{for}\;i=1,m=1\;\\ \alpha_{\mathrm{ims}}+\lambda_{\mathrm{ims}}T_{11s}+\delta_{\mathrm{ims}}O_{11\mathrm{ts}}+{\mathrm{TM}}_{\mathrm{ims}}+\gamma_{\mathrm{ims}}{\mathrm{OM}}_{\mathrm{mts}}+\varepsilon_{\text{imts}}\;\text{for}\;\left(i,m\right)\neq 1 \end{array}\right. \end{array}$$

where *α*_*ims*_ is a constant intercept and *λ*_*ims*_, *δ*_*ims*_, and *γ*_*ims*_ are factor loadings.

**TABLE 2:** Glossary of manifest and latent variables of the multimethod latent state–trait model for random and fixed situations.

Notation	Model component	Interpretation	Example
*Y*_*imts*_	Manifest variable	Observed score of indicator *i*, measured by method *m* on occasion *t* in fixed situation *s*	*Y*_2211_: Observed value for the offline, false‐keyed Depression item on occasion one
*T*_*11s*_	Reference indicator trait factor	Construct score/trait level—measured by the reference indicator and method^a^ (*i* & *m* = 1)—specific to fixed situation *s* but stable across occasions *t*	*T*_112_: Stable online trait level of Negative Emotionality, measured by the true‐keyed Anxiety item across both measurement occasions
*O*_*11ts*_	Reference indicator occasion factor	Occasion‐specific deviation^b^—measured by the reference indicator and method (*i* & *m* = 1)—from the stable trait level in fixed situation *s*	*O*_1121_: Deviation from general offline Negative Emotionality specific to occasion two measured by the true‐keyed Anxiety item
*TM*_*ims*_	Trait‐method factor	Trait‐like method effect for indicator *i—*measured by method *m* in fixed situation *s—*reflecting variance not shared with the reference indicator in fixed situation *s*	*TM*_321_: Response variability of the false‐keyed Volatility item in the offline context, which is stable across occasions but is not shared with the true‐keyed Anxiety item
*OM*_*mts*_	Occasion‐ method factor	Occasion‐specific method effect of method *m* in fixed situation *s*, reflecting variance not shared with the reference indicator on occasion *t*	*OM*_112_: Response variability of the true‐keyed non‐reference indicators for online Negative Emotionality on occasion one which is not shared with the true‐keyed Anxiety item
*ϵ*_*imts*_	Measurement error variable	Unexplained variance of indicator *i*, measured by method *m* on occasion *t* in fixed situation *s*	*ϵ*_2112_: Unique, unexplained variance for the true‐keyed Depression item on occasion one in the online context

*Note: i* = indicator, *m* = method, *t* = occasion, *s* = fixed situation.

^a^ In the model a reference indicator and method are chosen in relation to which the latent variables are defined and interpreted. In the motivational example, the Anxiety item is the reference indicator and true‐keyed items constitute the reference method.

^b^ Occasion factors (as well as trait‐method and occasion‐method factors) are defined as residuals to the trait factors. The deviation term is used to refer to a deviation from the predicted trait level and does not imply a difference score.

Given the complexity of MM‐LST‐RF models, we initially present two simpler LST model extensions that introduce key components of the full model: (a) the LST‐RF model captures fixed situation components and (b) the MM‐LST model captures multimethod components. These two simpler models provide a solid foundation for understanding the full MM‐LST‐RF model.

#### 1.6.1 The LST‐RF Model

The LST‐RF model (Geiser et al. 2015) is designed for longitudinal data involving multiple fixed situations. Its key premise is that a person's trait level may vary across these predefined situations, like Negative Emotionality trait levels differing between the offline and online context. This is formalized by specifying separate LST models for each fixed situation, where all observed variables *Y*_*its*_ are expressed as a function of a latent trait‐factor *T*_*s*_, occasion factor *O*_*ts*_, and measurement error *ϵ*_*its*_ (Equation 2).

(2) $$Y_{\mathrm{its}}=\alpha_{\text{is}}+\lambda_{\text{is}}T_{s}+\delta_{\text{is}}O_{\mathrm{ts}}+\varepsilon_{\mathrm{its}}$$

where *α*_*is*_ is a constant intercept and *λ*_*is*_ and *δ*_*is*_ are factor loadings. *α*_*is*_, *λ*_*is*_, and *δ*_*is*_ are all assumed to be invariant across time (i.e., they are the same for all measurement occasions).

The observed variables *Y*_*its*_ serve as indicators of the latent construct. These indicators can be individual items, item parcels, subscales, test halves, or similar measures. Each indicator *i* corresponds to a specific occasion *t* and fixed situation *s* and also contains a unique, unsystematic error component *ϵ*_*its*_. Each fixed situation has a trait‐factor *T*_*s*_, capturing the trait‐like construct that is stable across all measurement occasions within that fixed situation.^5^ These trait factors may correlate, reflecting the extent to which the latent construct generalizes across fixed situations. Occasion factors *O*_*ts*_ are defined as residuals of the trait factors and capture occasion‐specific deviations^6^ from the trait within a fixed situation. These occasion‐specific deviations may differ between fixed situations. In our example, this would, for instance, allow stronger occasion‐specific effects in either the offline or online context.

**FIGURE 1:**
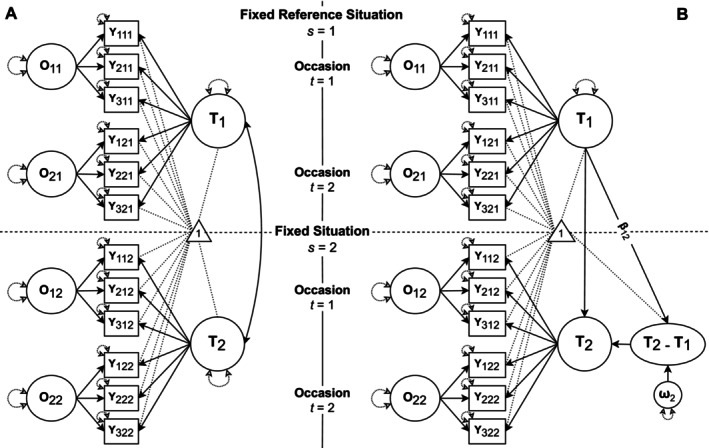
Two parametrizations of the LST‐RF model. (A) Model with correlated trait factors. (B) Model with latent difference variable (*T*_*2*_ − *T*_1_). The triangle represents the mean structure of the models. To improve readability, only the regression path *β*_12_ is labeled and only the residual variable *ω*_2_ is shown; error variables *ε*_*its*_ are not displayed. *T* = trait factors, *O* = occasion factors; *i* = indicator, *t* = occasion, *s* = fixed situation.

Figure 1A depicts a path diagram of an LST‐RF model. As the LST‐RF model does *not* accommodate multiple methods, we will illustrate it using only the true‐keyed items from our example dataset. Accordingly, the observed variables *Y*_*its*_ are NAt (*i* = 1), NDt (*i* = 2), and NVt (*i* = 3). *Y*_322_, for instance, refers to the observed score of the true‐keyed Volatility item NVt, measured at occasion two (*t* = 2) in the online context (*s* = 2). The trait factors *T*_1_ and *T*_2_ represent the general Negative Emotionality trait levels across both occasions for the offline (*s* = 1) and online context (*s* = 2) respectively. Correlations between *T*_1_ and *T*_2_ indicate cross‐context generalizability, where higher correlations suggest greater generalizability. The occasion factors *O*_*ts*_ capture deviations from the general Negative Emotionality score due to occasion‐specific influences. *O*_21_, for example, represents deviations from the trait‐level *T*_1_ in the offline context (*s* = 1) at occasion two (*t* = 2).

##### 1.6.1.1 Interaction Effects

To study person‐by‐fixed situation interactions, an alternative but equivalent parameterization is used. Paralleling the use of dummy variables in multiple regression, a fixed reference situation is specified (Eid 2000; Eid et al. 2008). This fixed situation is chosen as a comparison standard against which each of the remaining fixed situations can be contrasted. As this choice can influence parameter estimates (Maydeu‐Olivares and Coffman 2006), it should ideally represent a typical or neutral situation (Geiser et al. 2012, 2015). In our example, this would be the real world, which is the more typical out of the two contexts.

Based on the latent difference approach (Raykov 1993; Steyer et al. 1997), trait factors of fixed non‐reference situations (*s* ≠ 1) are decomposed into the reference trait *T*_1_ and a latent difference variable (*T*_*s*_ − *T*_1_) as shown in Equation (3).

(3) $$T_{s}=T_{1}+\left(T_{s}-T_{1}\right)$$

The latent difference variable *T*_*s*_ − *T*_1_ reflects differences^7^ in trait scores between fixed situations. Its mean indicates whether, on average, individuals provide different responses depending on the fixed situation. In our example, the mean of *T*_*s*_ − *T*_1_ is zero when there are no differences between the offline and online contexts, positive when scores are higher online, and negative when they are higher offline. The variance of *T*_*s*_ − *T*_1_ shows how much individuals differ in their responses across contexts. If all individuals react in the same manner, the variance is zero, and the trait factors are perfectly correlated (*Corr*(*T*_1_, *T*_*s*_) = 1). The more individuals vary in their reactions to the two contexts, the larger the variance of the difference variable becomes.

As illustrated in Figure 1B, the difference variable can be regressed on the reference trait (Equation 4) to estimate person‐by‐fixed situation interactions, following the latent change score modeling approach by McArdle (2009).

(4) $$\left(T_{s}-T_{1}\right)=\beta_{0s}+\beta_{1s}T_{1}+\omega_{s}$$

where *β*_0*s*_ is a constant intercept, *β*_1*s*_ is a regression slope coefficient, and *ω*_*s*_ represents unique variance that cannot be explained by the reference trait factor. In our example, a positive *β*_12_ implies a synergistic effect (Schmitt et al. 2003), where individuals with higher offline Negative Emotionality report larger differences between contexts. In contrast, a negative *β*_12_ implies a buffering effect (Cohen et al. 2003), where higher Negative Emotionality scores in the offline context correspond to smaller differences. A *β*_12_ value of zero implies that no linear person‐by‐fixed situation interaction is present.^8^ Additional predictors can be added to explain variability in the difference score. In our example, such variables could, for instance, be motivations for internet use, social anxiety, problematic internet use, or online self‐presentation.

In summary, LST‐RF models introduce the fixed situation component essential to the full MM‐LST‐RF model by capturing context‐specific traits and person‐by‐fixed situation interactions, while simultaneously accounting for occasion‐specific fluctuations and measurement error. We now turn to MM‐LST models, which address the multimethod component necessary for obtaining the full MM‐LST‐RF model.

#### 1.6.2 The MM‐LST Model

**FIGURE B1:**
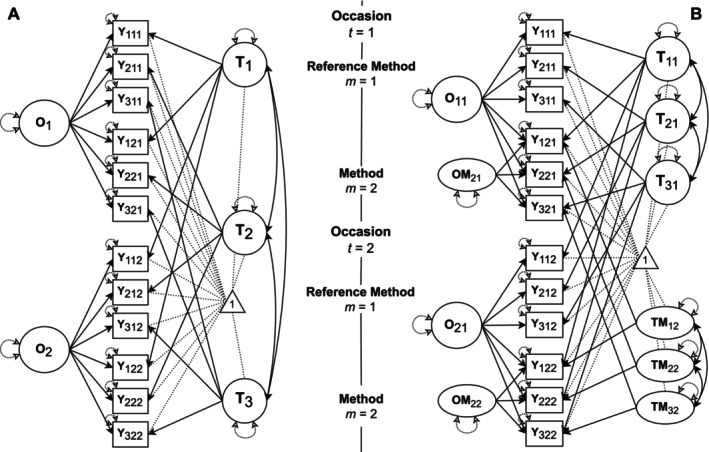
Derivation of an MM‐LST model using the reference method approach. (A) LST model without method factors. (B) MM‐LST model with method factors. The triangle represents the mean structure of the models. To improve readability, error variables ε_imt_ are not shown and paths are not labeled. *T* = trait factors, *O* = occasion factors, *TM* = trait‐method factor, *OM* = occasion‐method factor; *i* = indicator, *m* = method, *t* = occasion.

The MM‐LST model (Courvoisier 2006; Courvoisier et al. 2008) extends LST theory to handle multiple methods. It integrates LST models with correlated trait‐correlated method minus one models (Eid 2000; Eid et al. 2003), which can handle multitrait‐multimethod data. Following a reference method approach, one method is specified as a comparison standard, and the remaining method(s) are modeled as deviations from it (see Figure B1 in Appendix B).

The full MM‐LST‐RF model draws on this approach but uses a reference indicator instead of a reference method (Hintz et al. 2019). This involves selecting a single item to serve as a comparison standard. Since each indicator is measured by a specific method, selecting an indicator also implies choosing a reference method. The choice should either represent the most valid or accurate measure of the construct, or reflect an established standard (Eid 2000; Geiser et al. 2008, 2012). Since we present the MM‐LST model with the goal to foster understanding of the core concepts of MM‐LST‐RF models, we will follow the reference indicator approach in the remaining explanations.

Applying the reference indicator approach, we can distinguish between two types of observed variables, reference indicators and non‐reference indicators. Reference indicators *Y*_11*t*_ are typically modeled as the first indicator (*i* = 1) and the method they are assessed with as the first method (*m* = 1). They are a function of a latent trait‐factor *T*_11_, occasion factor *O*_11*t*_, and measurement error *ϵ*_*imt*_. Non‐reference indicators *Y*_*imt*_ (*i & m* ≠ 1) additionally measure trait‐method factors *TM*_*im*_ and occasion‐method factors *OM*_*mt*_ (Equation 5).

(5) $$\begin{array}{c} Y_{\mathrm{imt}}=\left\{\begin{array}{c} \;T_{11}+O_{11t}+\varepsilon_{11t}\;\text{for}\;i=1,m=1\;\\ \alpha_{\mathrm{im}}+\lambda_{\mathrm{im}}T_{11}+\delta_{\mathrm{im}}O_{11t}+{\mathrm{TM}}_{\mathrm{im}}+\gamma_{\mathrm{im}}{\mathrm{OM}}_{\mathrm{mt}}+\varepsilon_{\mathrm{imt}}\;\text{for}\;\left(i,m\right)\neq \left(1,1\right) \end{array}\right. \end{array}$$

where *α*_*im*_ is a constant intercept and *λ*_*im*_, *δ*_*im*_, and *γ*_*im*_ are factor loadings. The *α*_*im*_, *λ*_*im*_, *δ*_*im*_, and *γ*_*im*_ parameters are assumed to be time‐invariant.

**FIGURE 2:**
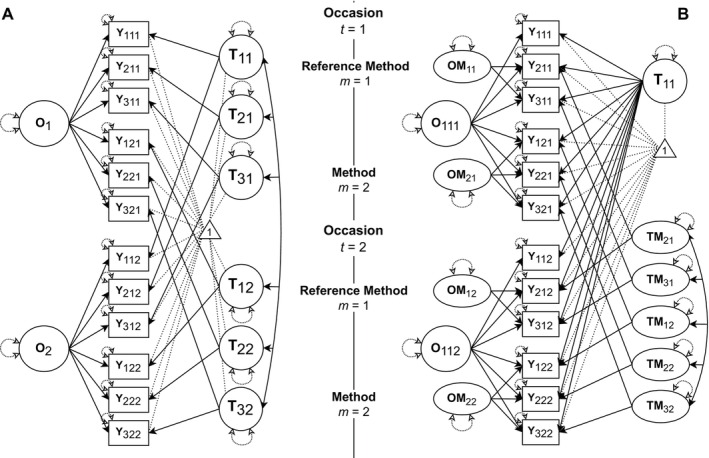
Derivation of a reference indicator MM‐LST model. (A) LST model without method factors. (B) MM‐LST model with method factors. The triangle represents the mean structure of the models. To improve readability, error variables *ε*_*imt*_ are not shown and paths are not labeled. *T* = trait factors, *O* = occasion factors, *TM* = trait‐method factor, *OM* = occasion‐method factor; *i* = indicator, *m* = method, *t* = occasion.

To better understand the individual parts of the MM‐LST model, we first examine a simplified model without method factors. Given that MM‐LST models only consider one fixed situation, we will only use the offline context from our example dataset. In the model shown in Figure 2A, trait factors *T*_*im*_ are both indicator‐ and method‐specific, reflecting that each item may measure the latent construct slightly differently. In our case, six trait factors are included—three for true‐keyed items (*m* = 1) and three for false‐keyed ones (*m* = 2). *T*_21_, for instance, represents the trait level of Negative Emotionality as measured by the true‐keyed Depression item across all occasions. Occasion factors *O*_*s*_ are defined as residuals to *T*_*im*_ and capture deviations from the trait factors shared by all indicators within a given occasion, regardless of method. In our example, there are two such factors, *O*_1_ and *O*_2_, corresponding to the two measurement occasions.

##### 1.6.2.1 Method Effects

To incorporate method factors, as shown in Figure 2B, a reference indicator—and thus a reference method—must be selected. This indicator's loading is fixed to one and its intercept to zero, which is why α_ims_, λ_ims_, and δ_ims_ are omitted for it in Equation (5). It defines the measurement unit and origin for the trait and occasion factors, highlighting the importance of choosing a suitable comparison standard. In our example, the true‐keyed Anxiety item serves as the reference indicator because it represents the most central facet of Negative Emotionality (Hofstee et al. 1992; Soto and John 2017b); as a true‐keyed item, it defines the positive direction of the construct. Under the assumption of time invariance, it serves as the reference indicator across all occasions.

With the reference point set, indicator‐ and method‐specific traits are reduced to a single common trait‐factor *T*_11_, representing Negative Emotionality as measured by the true‐keyed Anxiety item *Y*_11*t*_.^9^ Occasion factors *O*_11*t*_ remain occasion‐specific, but are now also defined by the reference item. *O*_111_ and *O*_112_ therefore reflect the deviation from *T*_11_ on occasions one and two respectively, as measured by the true‐keyed Anxiety item.

Two types of method factors are then introduced: trait‐method factors *TM*_*im*_ and occasion‐method factors *OM*_*mt*_. These method factors apply only to *non*‐reference indicators; they represent systematic deviations from *T*_11_ attributable to the method. Trait‐method factors capture systematic response variability that is specific to each indicator but stable across occasions. In our example, five trait‐method factors exist—two for true‐keyed (NAt, NDt) and three for false‐keyed items (NVf, NAf, NDf). *TM*_32_, for instance, reflects variance in the false‐keyed Volatility item not shared with true‐keyed Anxiety. These factors may correlate, reflecting common method effects across indicators; low correlations suggest method‐specific effects, whereas high correlations suggest generalization.

Occasion‐method factors capture systematic, occasion‐specific deviations from *T*_11_ shared by all non‐reference indicators of the same method at a given occasion. In contrast to trait‐method factors, they are *not* allowed to correlate. In our case, four occasion‐method factors exist, one for each combination of method and occasion. For instance, *OM*_21_ captures shared variance among false‐keyed items at the first occasion, which is not shared with true‐keyed Anxiety. This reflects the possibility that methods may be differently affected by occasion‐specific influences. As an example, distressing world news could have negatively impacted participants' moods on the first occasion, leading them to respond more strongly to negatively worded, mood‐congruent items (e.g., “*worried*,” “*depressed*”) than to positively worded, incongruent ones (e.g., Neumann et al. 2001). Consequently, true‐ and false‐keyed items would have been affected differently at occasion one but not at occasion two.

In summary, MM‐LST models address methods effects by differentiating among five sources of variance: trait, occasion, and error (as in classic LST theory), plus trait‐method and occasion‐method variance. However, they do not address multiple fixed situations.

### 1.7 Combining the Models

**FIGURE 3:**
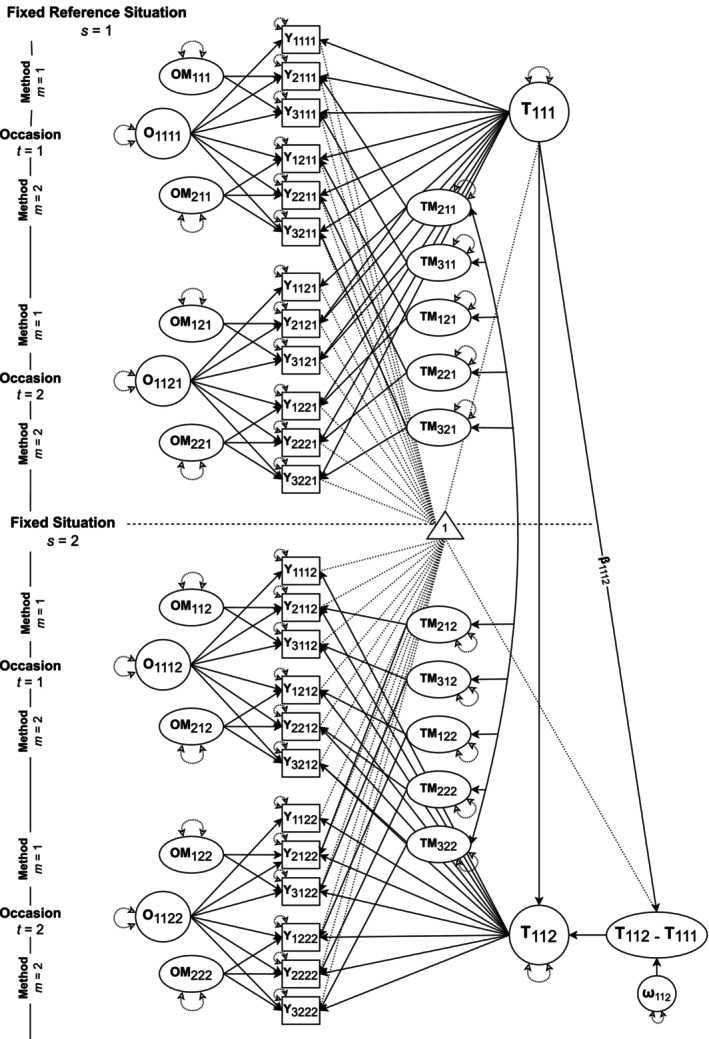
MM‐LST‐RF model with a latent trait difference variable (*T*_112_ − *T*_111_). The triangle represents the mean structure of the models. To improve readability, only the regression path *β*_1112_ is labeled and only the residual variable *ω*_112_ is shown; error variables *ε*_*imts*_ are not displayed. *T* = trait factors, *O* = occasion factors, *TM* = trait‐method factor, *OM* = occasion‐method factor; *i* = indicator, *m* = method, *t* = occasion, *s* = fixed situation.

**FIGURE B2:**
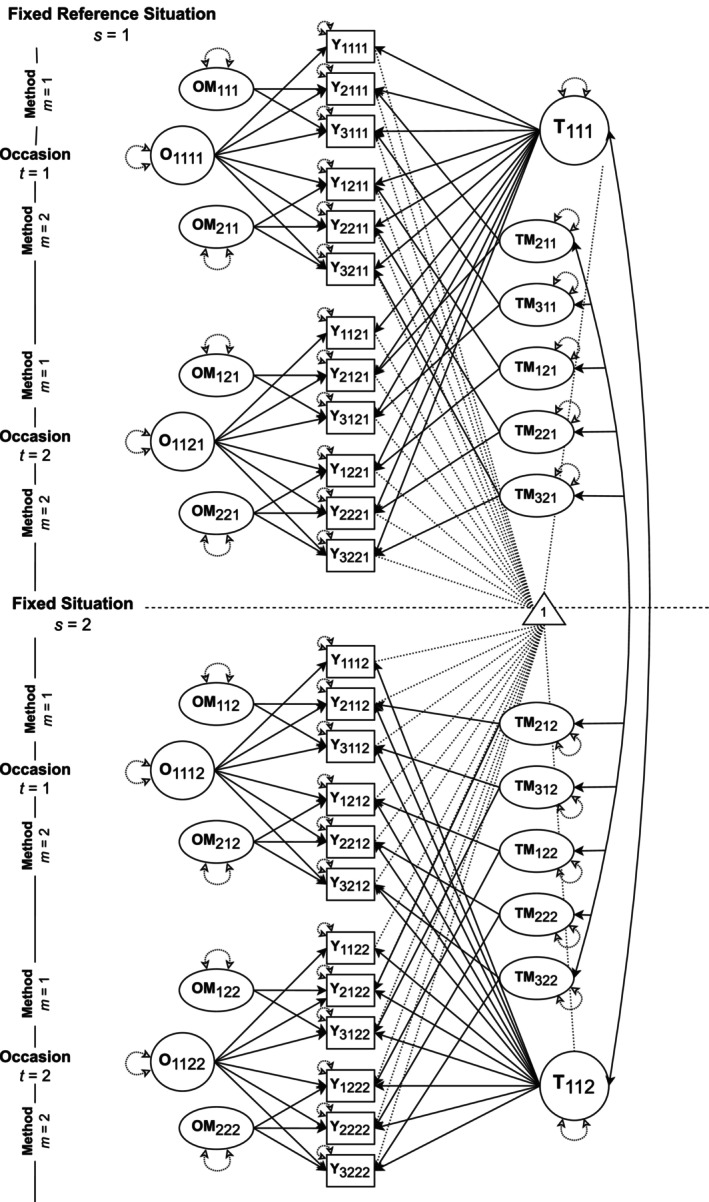
MM‐LST‐RF model with correlated trait factors. The triangle represents the mean structure of the models. To improve readability, error variables ε_*imts*_ are not shown and paths are not labeled. *T* = trait factors, *O* = occasion factors, *TM* = trait‐method factor, *OM* = occasion‐method factor; *i* = indicator, *m* = method, *t* = occasion, *s* = fixed situation.

Combining the two LST extensions—the LST‐RF and MM‐LST models—yields the full MM‐LST‐RF model (Hintz et al. 2019), which accounts for both multiple methods and fixed situations. The integration of the two models is achieved by specifying the more comprehensive MM‐LST model for each fixed situation, instead of a simple LST model. Figure 3 illustrates an MM‐LST‐RF model for our motivational example. As with LST‐RF models, MM‐LST‐RF models can either be specified with a latent trait difference variable (as in Figure 3) or with correlated trait factors (see Figure B2 in Appendix B).

Coming back to the initially presented Equation (1), the combination of LST‐RF and MM‐LST models becomes clear. It extends Equation (5) from MM‐LST models by implementing the fixed situations introduced in LST‐RF models. All model components are now fixed situation‐specific, as indicated by the subscript *s*. As in MM‐LST models, each fixed situation has a reference indicator, which defines the measurement origin and unit for the trait and occasion factors. Accordingly, there are two measurement equations: one for the reference indicators and one for non‐reference indicators (Equation 1).

(1 repeated) $$\begin{array}{c} Y_{\text{imts}}=\left\{\begin{array}{c} \;T_{11s}+O_{11\mathrm{ts}}+\varepsilon_{11\mathrm{ts}}\;\text{for}\;i=1,m=1\;\\ \alpha_{\mathrm{ims}}+\lambda_{\mathrm{ims}}T_{11s}+\delta_{\mathrm{ims}}O_{11\mathrm{ts}}+{\mathrm{TM}}_{\mathrm{ims}}+\gamma_{\mathrm{ims}}{\mathrm{OM}}_{\mathrm{mts}}+\varepsilon_{\text{imts}}\;\text{for}\;\left(i,m\right)\neq 1) \end{array}\right. \end{array}$$

where *α*_*ims*_ is a time‐invariant intercept and *λ*_*ims*_, *δ*_*ims*_, and *γ*_*ims*_ are time‐invariant factor loadings. Intercepts and loadings of the reference indicator are fixed (*α*_*ims*_ = 0, *λ*_*ims*_ & *δ*_*ims*_ = 1) and do not appear in Equation (1). The measurement equation of non‐reference indicators additionally incorporates trait‐method factors *TM*_*ims*_ as well as occasion‐method factors *OM*_*mts*_ which account for potential measurement deviations attributable to the employed non‐reference methods. Loadings on trait‐method factors do not appear in the equation because they are all fixed to one to ensure their identification and maintain time invariance. To identify the occasion‐method factors, one *γ*_*ims*_ must be fixed to one. No further constraints on *λ*_*ims*_ or *δ*_*ims*_ are required.

#### 1.7.1 Person‐ and Method‐by‐Fixed Situation Interaction Effects

Person‐by‐fixed situation interaction effects can be estimated just like in LST‐RF models. First, a fixed reference situation is selected. Then, the difference variables (*T*_1*s*_ − *T*_111_) between the non‐reference and reference traits are calculated (Equation 6) and regressed on the reference trait *T*_111_ (Equation 7).

(6) $$T_{11s}=T_{111}+\left(T_{11s}-T_{111}\right)$$

(7) $$\left(T_{11s}-T_{111}\right)=\beta_{011s}+\beta_{111s}T_{111}+\omega_{11s}$$

where *β*_011*s*_ is a constant intercept, *β*_111*s*_ a regression slope, and *ω*_11*s*_ the residual variance of the difference variable. Table 3 summarizes these coefficients and their interpretations with examples from our illustrative data.

**TABLE 3:** Latent change score model components.

Notation	Model component	Interpretation	Example
(*T*_11*s*_ − *T*_111_)^a^	Trait‐factor difference variable	Differences between the trait level of a fixed non‐reference situation and the reference situation (*s* = 1)	(*T*_11*2*_ − *T*_111_): Difference between the Negative Emotionality trait levels in the online and offline context
(*TM*_*ims*_ − *TM*_*im1*_)^a^	Trait‐method factor difference variable	Differences in trait‐like method effects between a fixed non‐reference situation and the reference situation (*s* = 1)	(*TM*_*322*_ − *TM*_*321*_): Difference between the trait‐like method effect of the false‐keyed Volatility items in the online and offline context
β_011s_	Intercept coefficient^b^	Expected situation effect when the reference situation (*s* = 1) trait level is zero	*β*_*0112*_: Expected situation effect when the offline Negative Emotionality score is zero
β_111s_/β_1ims_ ^c^	Regression coefficient	Indicator of linear person‐/method‐by‐fixed situation interaction effect	*β*_*1112*_: Interaction effect between Negative Emotionality and context (offline & online)^d^
ω_1ms_/ω_ims_ ^c^	Latent residual of difference variable	Unique situation effect not predicted by the level of the trait/trait‐method factor in the fixed reference situation (*s* = 1)	*ω*_*112*_: Unique situation effect in the online context which cannot be predicted by offline Negative Emotionality^d^

*Note: i* = indicator, *m* = method, *t* = occasion, *s* = fixed situation.

^a^ *i* = 1 & *m* = 1.

^b^ There is no intercept coefficient for the trait‐method difference variable as trait‐method factors are defined as residuals with means of zero.

^c^ Left of slash = trait‐factor change score parameter; right of slash = trait‐method factor change score parameter.

^d^ Example shown for latent trait‐factor change score model.

The same logic can be applied to trait‐method factors to estimate method‐by‐fixed situation interaction effects. Trait‐method factors from fixed non‐reference situations are compared to their counterparts in the fixed reference situation, yielding the latent difference variable (*TM*_*ims*_ − *TM*_*im*1_) shown in Equation (8). This reflects the assumption that method effects can vary across contexts.

(8) $${\mathrm{TM}}_{\mathrm{ims}}={\mathrm{TM}}_{\mathrm{im}1}+\left({\mathrm{TM}}_{\mathrm{ims}}-{\mathrm{TM}}_{\mathrm{im}1}\right)$$

**FIGURE B3:**
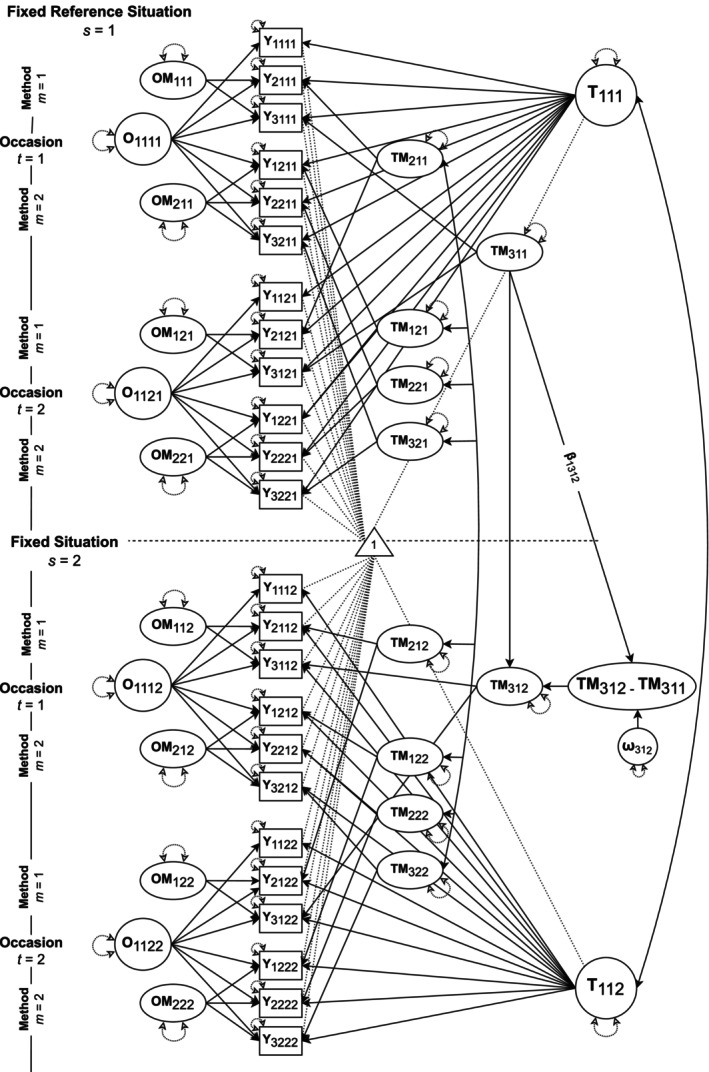
MM‐LST‐RF model with a latent trait‐method factor difference variable (*TM*_312_ − *TM*_311_). The triangle represents the mean structure of the models. To improve readability, only the regression path β_312_ is labeled and only the residual variable ω_1312_ is shown; error variables ε_*imts*_ are not displayed. *T* = trait factors, *O* = occasion factors, *TM* = trait‐method factor, *OM* = occasion‐method factor; *i* = indicator, *m* = method, *t* = occasion, *s* = fixed situation.

The difference variable is then regressed on the corresponding trait‐method factor of the fixed reference situation (Equation 9).

(9) $$\left({\mathrm{TM}}_{\mathrm{ims}}-{\mathrm{TM}}_{\mathrm{im}1}\right)=\beta_{1\mathrm{ims}}{\mathrm{TM}}_{\mathrm{im}1}+\omega_{\mathrm{ims}}$$

where *β*_1*ims*_ represents the regression slope and *ω*_*ims*_ the residual variance (see also Table 3). Since trait‐method factors are defined as residual factors, no intercept is included. A nonzero regression coefficient suggests that differences in method effects (with means = 0) across fixed situations depend on the method effect in the reference situation. For instance, a highly positive *β*_1322_ indicates that a strong method effect in the offline context predicts larger online‐offline discrepancies for the false‐keyed Volatility item. Figure B3 in Appendix B shows a path diagram including a trait‐method factor difference variable.

#### 1.7.2 Coefficients

Beyond the discussed parameters, several coefficients address key questions within the MM‐LST‐RF model. Three indicator‐specific coefficients originating from classic LST models also apply to MM‐LST‐RF models (see Table A1 in Appendix A for formulas):

- *Consistency Con*(*Y*_*imts*_) represents the proportion of an indicator's variance explained by the trait factor (i.e., the stable trait variance shared across occasions). Larger values imply more consistent indicators. While trait‐method factors also explain stable variance, they are excluded from consistency calculations since they represent method‐specific variance.
- *Specificity Spe*(*Y*_*imts*_) captures the portion of variance that is attributable to occasion factors that fluctuate across measurement occasions. Higher specificity implies that indicators are more sensitive to occasion‐specific influences. Again, method‐specific variance explained by occasion‐method factors is excluded from this coefficient, as it is method specific.
- *Reliability Rel*(*Y*_*imts*_) reflects the total explainable variance of an indicator, encompassing trait, occasion, and method factors. Indicators with higher reliability provide more accurate estimates of the underlying latent variables.^10^

MM‐LST‐RF models offer additional coefficients not available in classic LST models. We highlight four that are central to addressing key questions in multi‐situation and multimethod research (see Table A1 in Appendix A for formulas). Additional coefficients are discussed in Hintz et al. (2019, 10–11), but are omitted here to keep the tutorial accessible.

- *Commonality of trait factors Comm*(*T*_11*s*_): The correlation of trait factors *Corr*(*T*_111_, *T*_11*s*_) indicates to what degree the latent construct generalizes across fixed situations. Squaring the correlation provides a measure of trait consistency, with higher values indicating a larger amount of shared variance between trait factors of different fixed situations.
- *Fixed situation specificity of trait factors SitSpe*(*T*_11*s*_): The complement of commonality reflects the extent to which trait factors are unique to each fixed situation. It is calculated as 1−*Comm*(*T*_11*s*_), with higher values indicating a stronger influence of the fixed situations.
- *Commonality of trait‐method factors Comm*(*TM*_*ims*_): Similar to the commonality of trait factors, this coefficient measures the consistency of trait‐method factors across fixed situations by squaring the correlation between corresponding trait‐method factors [*Corr*(*TM*_*im*1_, *TM*_*ims*_)]^2^.
- *Fixed situation specificity of trait‐method factors SitSpe*(*TM*_*ims*_): This coefficient complements the commonality of trait‐method factors by quantifying the extent to which these factors vary across fixed situations, thereby capturing their situation specificity.

These coefficients help researchers assess the quality and generalizability of measurements in multimethod and multi‐context designs. With this foundational understanding of the MM‐LST‐RF model in place, we can now apply MM‐LST‐RF models to our motivational example and explore the practical decisions involved in model specification and estimation using our shiny app.

## 2 Shiny App Tutorial

In the second part of our paper, we address practical challenges in estimating MM‐LST‐RF models. Due to the complexity and length of the required syntax, we developed a new function and shiny app to automate **lavaan** (Rosseel 2012) code generation and model estimation. Both tools allow users to define model restrictions and access detailed model summaries. The shiny app offers a more accessible interface for researchers less familiar with R, enabling analysis without directly using the **mmLSTrf()** function in R. It supports most standard use cases, with only minor limitations noted throughout this tutorial. In comparison, directly using the **mmLSTrf()** function makes it easier to document and automate analyses, thereby enhancing reproducibility. It provides access to the underlying code, allows for greater customization, and facilitates integration with other **lavaan**‐based R packages. It simplifies model comparisons and supports more advanced **lavaan** functionality. A detailed manual for the **mmLSTrf()** function is available in the Supplement. The following sections guide users step‐by‐step through estimating an MM‐LST‐RF model in the shiny app, using our motivational example.

**Step 1: Launching the app**.

To use the shiny app, the **lsttheory** package (Mayer 2025) needs to be installed and loaded in R before the graphical user interface can be launched (see code below).

**install.packages(“devtools”)**

**devtools::install_github(“amayer2010/lsttheory”)**

**library(lsttheory)**

**mmLSTrfGUI()**

**FIGURE 4:**
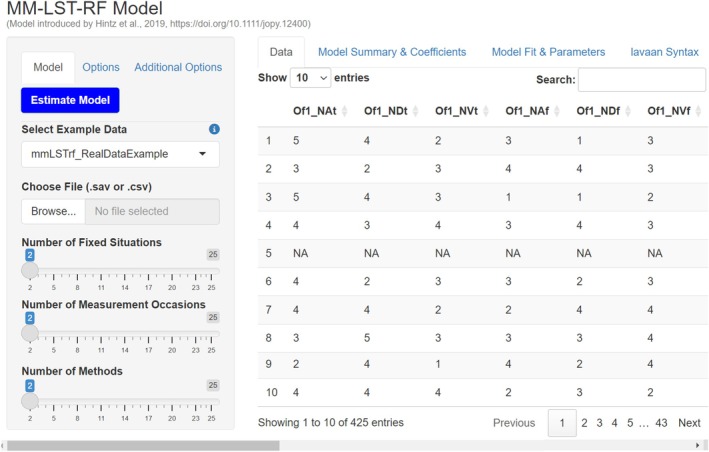
mmLSTrf shiny app interface with loaded data from the motivational example.

The interface includes two main panels, which are displayed in Figure 4. The smaller panel on the left has three tabs—*Model*, *Options*, and *Additional Options*—as well as an *Estimate Model* button. These tabs let users specify the desired MM‐LST‐RF model. Hovering the mouse over the blue “**i**” icons throughout the app provides additional guidance, explaining the purpose and effects of each option. The larger right panel displays outputs across four tabs: *Data*, *Model Summary & Coefficients*, *Model Fit & Parameters*, and *lavaan Syntax*.

**Step 2: Loading the data**.

Once the app has been launched, users can load the data. Two example datasets are integrated in the package and can be accessed via the *Select Example Data* drop‐down menu. In Figure 4 the *“mmLSTrf_RealDataExample”* is selected. It contains the dataset (*N* = 425) from our motivational example, consisting of the three true‐ and three false‐keyed items measuring Negative Emotionality at two occasions across offline and online contexts, resulting in 24 variables. Details about the items and variable names are in provided Table 1 or directly in R via? **mmLSTrf_RealDataExample**.

The second dataset, “*mmLSTrf_SimulatedDataExample*,” simulates a sample of *N* = 500 for an MM‐LST‐RF model with three indicators, two methods, three occasions, and two fixed situations. Compared to the real data example, it features an additional occasion, enabling exploration of more complex models. This simulated dataset is also less prone to estimation issues, providing an opportunity to experiment with various model restrictions and assess their effects. More details on this dataset are available via **?mmLSTrf_SimulatedDataExample** in R and in the Supplement.

To use one's own data, the user can upload .*csv* or .*sav* files through the *Browse*… button under the *Choose File* menu. As the app runs locally, no sensitive data is transmitted externally. Although there are no requirements for variable names, the loaded dataset must follow three structural rules. (1) The dataset should only contain the indicator variables required for the MM‐LST‐RF model; any irrelevant variables should be removed beforehand. (2) The dataset must be in wide format, with one row per participant and repeated responses across methods, occasions, or fixed situations placed in separate columns. (3) The variable order must follow the top‐to‐bottom indicator order illustrated in Figure 4, grouped first by fixed situations, then occasions, and then methods. In our example, the first half of the variables corresponds to the offline and the second half to the online context. Within each context, items from the first measurement occasion are listed before those from the second. Finally, within each occasion, the first three indicators are true‐keyed, while the last three are false‐keyed. Additional guidance is available in the function documentation accessible directly in R via **?mmLSTrf**.

Once loaded, users can verify the correct variable order and completeness of their data in the *Data* tab. Figure 4 displays the first six variables of our dataset, with additional variables accessible via the horizontal scroll bar at the bottom. The sample size appears just above the scrollbar (*N* = 425). By default, the first 10 entries are shown, with additional entries accessible through the page selection tool below the table. In our example, items were rated on a 5‐point Likert scale from “*disagree strongly*” [1] to “*agree strongly*” [5], with missing data displayed as “*NA*.” This becomes relevant later for specifying how missing data should be handled in model estimation.

**Step 3: Specifying the data structure**.

After loading the data, users must define the number of fixed situations, occasions, and methods using the sliders in the *Model* panel. The app supports up to 25 of each, slightly limiting its range compared to the **mmLSTrf()** function. In our example, we have two methods, two occasions, and two fixed situations. The number of indicators per method need not be specified, as it is assumed to be equal across methods and calculated automatically.

**Step 4: Applying model restrictions**.

**FIGURE 5:**
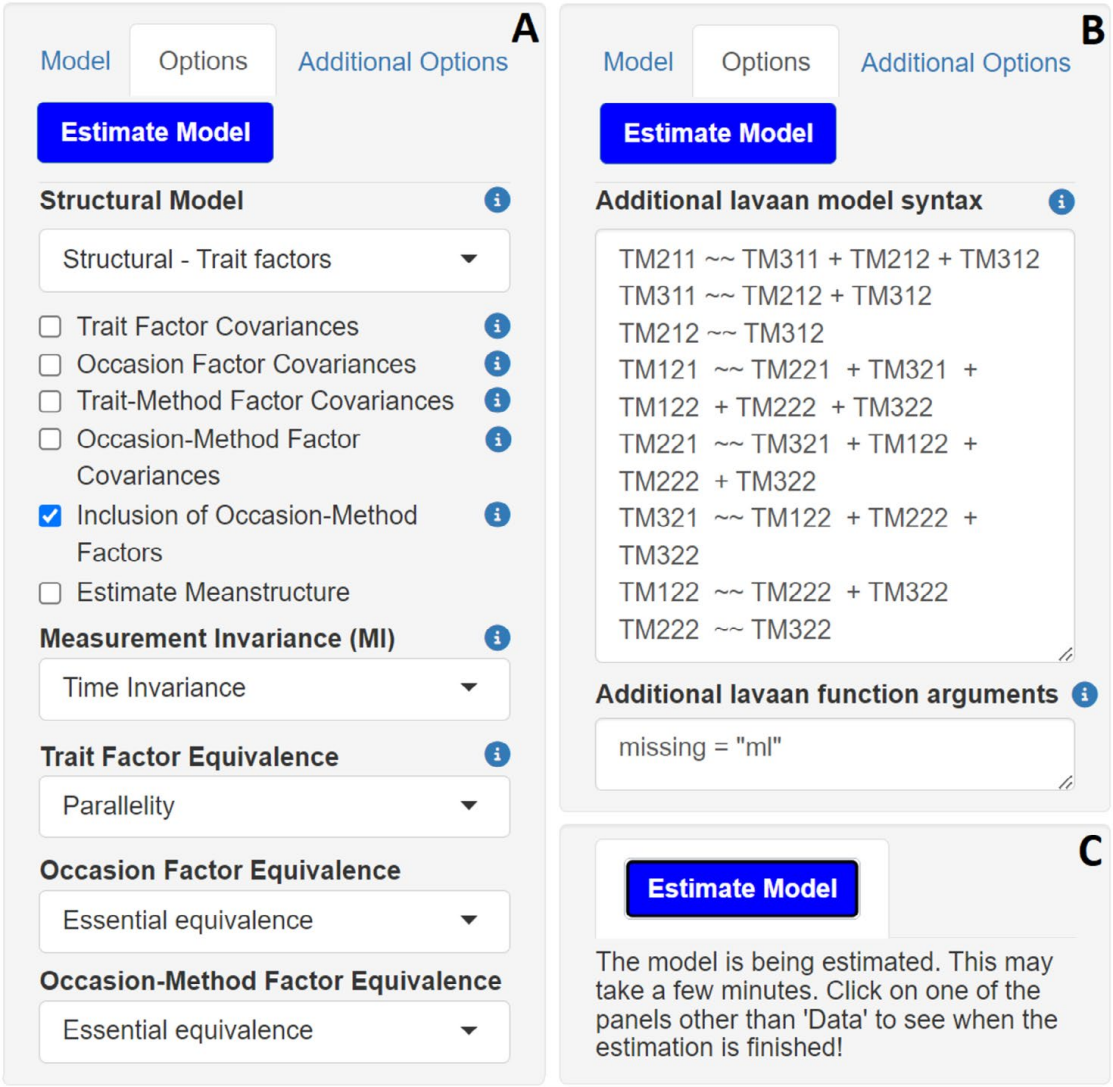
mmLSTrf shiny app input panels. (A) *Options* panel with default settings for specifying model restrictions. (B) *Additional Options* panel for custom lavaan syntax or arguments with example input. (C) Model estimation button.

Next, users specify model restrictions—crucial for parsimony, estimation, and interpretability—via the *Options* and *Additional Options* panels shown in Figure 5. The settings displayed in panel A reflect the function's default values. The following restrictions can be applied:

### 2.1 Structural Model

In the *Structural Model* drop‐down menu, users can choose to estimate person‐by‐fixed situation effects (*Structural—Trait factors*), method‐by‐fixed situation effects (*Structural—Trait‐Method factors*), or both. Selecting either option estimates all possible interaction effects of the chosen type. Person‐by‐fixed situation interaction effects will thus be estimated for each fixed non‐reference situation, and method‐by‐fixed situation interaction effects will be estimated for each trait‐method factor specific to fixed non‐reference situations. To omit interaction effects or specify custom ones, users can estimate the model without interaction effects (*Measurement model*) and enter custom syntax using the *Additional lavaan model syntax* input field in the *Additional Options* tab (Figure 5B). In our case, we want to examine person‐by‐fixed situation interaction effects, so we select the *Structural—Trait factors* option. Since there is only one fixed non‐reference situation in the empirical example—the online context—a single interaction effect will be estimated.

### 2.2 Covariances

Users can optionally estimate covariances among latent variables using the four checkboxes below the *Structural Model* menu. By default, all latent variables are uncorrelated. Selecting an option adds all admissible covariances to the model.^11^ Trait factors and trait‐method factors will correlate with all other factors of the same type, while occasion and occasion‐method factors will correlate with their counterparts in other fixed situations. Because too many covariances can make estimation more difficult, especially for trait‐method factors, users should use these options sparingly. Although including trait‐factor covariances is generally appropriate when no interactions are requested, other covariances should only be estimated with theoretical justification. For instance, covariances between occasion or occasion‐method factors are usually only reasonable if fixed situations are assessed close in time (Hintz et al. 2019).

Like interaction effects, custom covariances can also be added using custom syntax. In our example, it is reasonable to assume that occasion factors are correlated across contexts, since both fixed situations were assessed on the same occasions. Similarly, trait‐method factors of the same measurement method (i.e., true‐ or false‐keyed items) are likely correlated with each other. Accordingly, we selected the *Occasion Factor Covariances* option and manually specified the expected trait‐method factor correlations, as shown in Figure 5B.

### 2.3 Occasion‐Method Factors

The *Inclusion of Occasion‐Method Factors* option allows users to choose between a full MM‐LST‐RF model or a simplified version excluding occasion‐method factors since they may be fairly small in practice, which can add unnecessary complexity and reduce model performance (Hintz et al. 2019). By default, they are included. In our example, estimating them leads to negative variances, suggesting that occasion‐specific method variances are either nonexistent or very small. As they are not directly relevant to our research questions and we have no theoretical reason to expect them to be present in our example, we deselected this option to achieve admissible model solutions.

### 2.4 Estimate Mean Structure

This option is deselected by default. When ticked, it estimates a mean structure for the model. This feature is required for mean comparisons. Since we want to compare trait levels of Negative Emotionality across contexts, we selected this option.

### 2.5 Measurement Invariance

Measurement invariance testing evaluates whether a scale or test measures the same construct across different conditions, such as cultures, countries, languages, age groups, methods, or occasions (Horn and McArdle 1992; Leitgöb et al. 2023; Meredith 1993; Millsap 2011). Measurement invariance becomes critical when fundamental differences across groups, methods, or measurement occasions may influence how participants interpret and respond to items. Without measurement invariance, comparisons of correlations or means across conditions may be biased, limiting the validity and generalizability of findings (e.g., Boer et al. 2018; Putnick and Bornstein 2016).

Four levels of measurement invariance are typically distinguished: configural, metric (weak), scalar (strong), and residual (strict), each imposing increasingly strict constraints on model parameters (Putnick and Bornstein 2016; Widaman and Reise 1997). Configural invariance, the least restrictive, requires only that the factor structure (i.e., pattern of loadings) is the same across groups. Descriptively, this means that the underlying construct can be measured using the same indicators across groups. However, meaningful parameter comparisons require stricter levels of measurement invariance. Metric invariance assumes equal loadings, meaning each item contributes similarly to the latent construct across conditions, allowing comparisons of latent variable *relationships* (e.g., correlations, regressions). Scalar invariance adds equality of intercepts, making mean comparisons meaningful by ensuring differences in the latent variables reflect true mean differences rather than measurement bias. Residual invariance, the most restrictive level, additionally assumes equal residual variances, implying that group differences are entirely due to the latent constructs and not differences in measurement error (Leitgöb et al. 2023; Putnick and Bornstein 2016).

In MM‐LST‐RF models, measurement invariance can be tested across methods, occasions, and fixed situations. By default, these models do not impose equality constraints across methods or fixed situations, aside from structural comparability (configural invariance). However, scalar invariance across measurement occasions is assumed (i.e., time invariant loadings and intercepts). Accordingly, the shiny app's default is *Time Invariance*. Users can impose more restrictive levels of invariance across methods, fixed situations, or both via the *Measurement Invariance* drop‐down menu.

If full invariance at a given level is not supported, partial invariance can be tested by freeing specific parameters. This can be done by selecting a less restrictive invariance level and adding constraints manually via the *Additional lavaan model syntax* input field. Partial invariance is generally acceptable when most items remain invariant, but as the number of non‐invariant items increases, so does the risk of bias (Chen 2008; Steinmetz 2013). Researchers should thus carefully evaluate the extent of non‐invariance and its potential impact on their findings.

In our example, we aim to compare mean trait levels of Negative Emotionality across the offline and online contexts. To ensure these comparisons are meaningful, at least scalar invariance across fixed situations must be established. This is tested by estimating models with increasingly restrictive invariance constraints and comparing them, as described in Step 5 of this guide.

### 2.6 Equivalence Assumptions

While measurement invariance tests whether parameters remain stable across conditions (e.g., occasions, methods, fixed situations), equivalence focuses on the uniformity of parameters within a set of indicators measuring the same latent factor. Different levels of equivalence can be distinguished depending on whether restrictions are placed on loadings, intercepts, or error variances. The *Equivalence Assumption* options allow users to select between five levels of equivalence based on classical test theory (Lord and Novick 1968): congenericity, essential equivalence, equivalence, essential parallelity, and parallelity.

The least restrictive level is congenericity, which requires only identification‐related constraints: the first indicator of each latent variable has its loading fixed to one, and intercepts fixed to zero in the case of trait factors. These constraints ensure model identifiability without assuming specific measurement structures. For trait and occasion factors, the constraints apply by default due to them being defined by the reference indicators. In the case of trait‐method factors, the combination of these constraints and the assumption of time invariance leads to all loadings being fixed to one, eliminating the need for additional equivalence constraints. Given congenericity, items can differ in how they relate to the latent factors, allowing for variation in item difficulty, discrimination, and reliability.

The choice among the four increasingly restrictive equivalence levels beyond congenericity largely depends on theoretical considerations and how the test or items were constructed—such as whether the items were designed to be interchangeable or to reflect uniform scaling. Essential equivalence assumes equal loadings for all indicators of a latent variable, effectively fixing them to one due to the identification constraints.^12^ This assumption is appropriate when items are designed to be equally strong indicators of the construct and can be applied to trait, occasion, and occasion‐method factors. Further constraints apply only to trait factors, since occasion and occasion‐method factors are residuals with means fixed to zero. The level of equivalence additionally assumes equal intercepts—thus fixing them to zero—implying that the expected item scores are identical at any given level of the latent trait. Essential parallelity assumes equal loadings and error variances but allows for varying intercepts, reflecting equal reliability despite baseline differences. Parallelity, the most restrictive assumption, imposes equal loadings, intercepts, and error variances, implying that all items function identically. The equivalence level of parallelity can be appropriate for standardized scales with carefully calibrated items.

In practice, the appropriate level of measurement equivalence should be guided by both theoretical justifications and empirical evidence related to test design and construct interpretation. If there are no theoretical constraints guiding model specification, users are encouraged to begin with the most restrictive level of equivalence for each factor and relax it if needed. This is reflected in the shiny app defaults, where trait factors are set to *Parallelity* and all other latent variables to *Essential Equivalence*. If the model fit is poor, constraints can be gradually relaxed, and models compared (see Step 5 of this guide). For the data in our empirical example, testing more restrictive levels of equivalence—though not required by our research questions—resulted in inadmissible solutions. We therefore retained the *Congenericity* setting for all latent variables.

### 2.7 Additional Lavaan Model Syntax

Users can manually enter custom **lavaan** syntax—such as interaction terms, covariances, or loading patterns—using this input field. The entered syntax should follow **lavaan** conventions, with one specification per line. Syntax conventions used for the model specifications can be found in the Supplement as well as the function documentation accessible in R via **?mmLSTrf**. Notably, manual entries override conflicting selections in the shiny app. In our example, we passed covariances between trait‐method factors of indicators belonging to the same method to the argument (see Figure 5B).

### 2.8 Additional Lavaan Function Arguments

This field accepts additional standard arguments from the **lavaan** package (e.g., *estimator =* “*MLR*,” *missing* = “*ML*,” or *se* = “*robust*”). If multiple arguments are entered, commas need to be used to separate them. Because there are missing values in our dataset, we specify *missing* = “*ML*,” instructing the app to use FIML for model estimation.

**Step 5: Estimating the model**.

**FIGURE 6:**
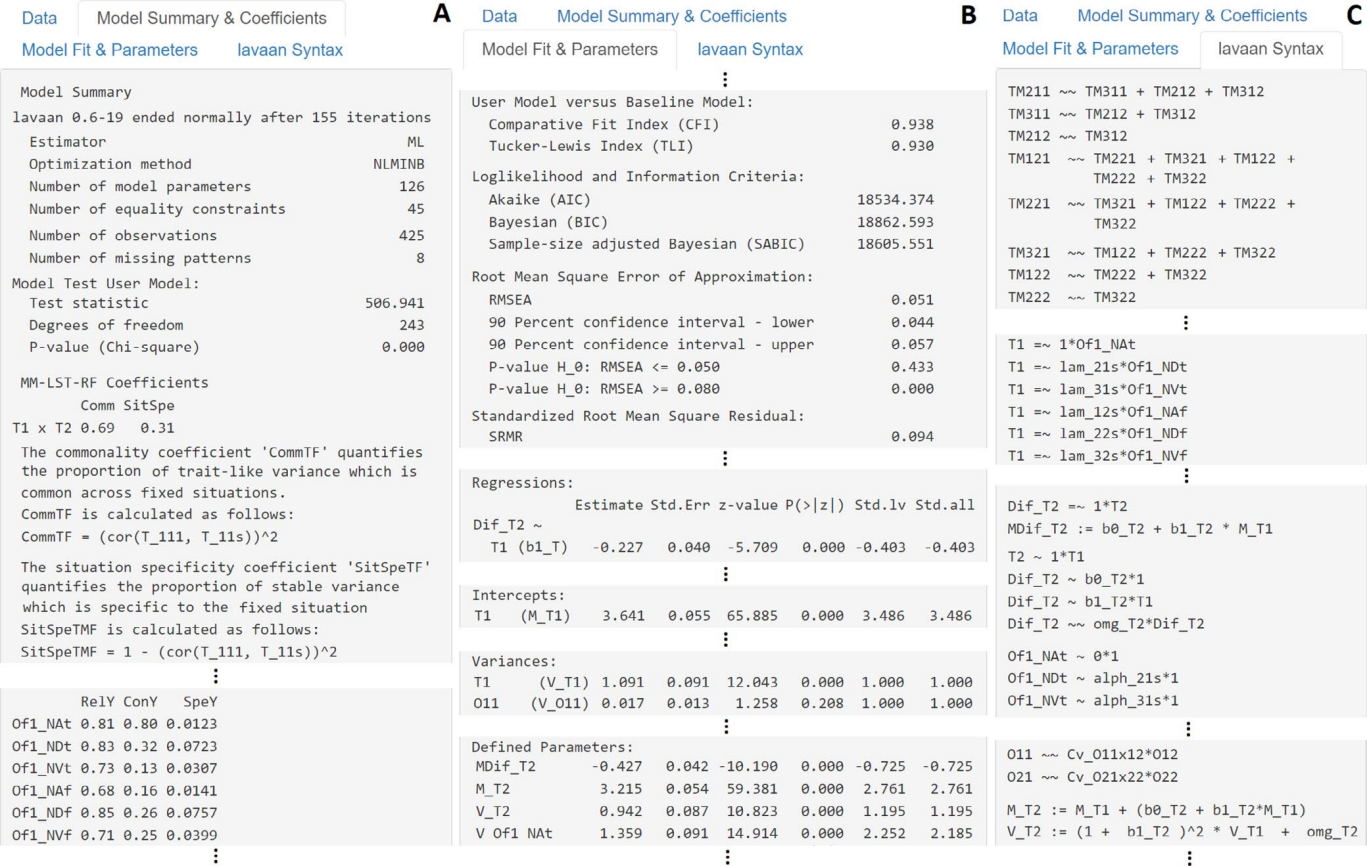
mmLSTrf shiny app model output panels displaying results from motivational example. (A) The *Model Summary & Coefficients* panel summarizes the estimated model and provides model coefficients. (B) The *Model Fit & Parameters* panel contains standard model fit indices and estimated parameters. (C) The *lavaan Syntax* panel shows the automatically generated syntax.

Once model specifications are finalized, the model is estimated by clicking the blue *Estimate Model* button (see Figure 5C). Unless specified otherwise under *Additional lavaan function arguments*, the app uses default settings from **lavaan::sem()**. Estimation may take several minutes depending on model complexity. If model estimation fails, the app will display a red error message indicating the cause. Warnings during estimation will appear separately in a yellow pop‐up notification. Once complete, results can be explored in the three output tabs *Model Summary & Coefficients*, *Model Fit & Parameters*, and *lavaan Syntax* (see Figure 6).

Panel A of Figure 6, titled *Model Summary & Coefficients*, provides the usual **lavaan** output, including the estimator used, number of free parameters, sample size, and the *p*‐value of the *χ* ^2^‐test statistic. It also contains the MM‐LST‐RF model coefficients, along with information regarding their calculation and interpretation. Panel B, *Model Fit & Parameters*, presents model fit indices as well as the parameter estimates resulting from the estimated model. Finally, Panel C, *lavaan Syntax*, displays the automatically generated model syntax, allowing users to verify that latent variables and restrictions were correctly implemented. This syntax can also be copied and used for further customization directly in R.

In our example, we aimed to compare Negative Emotionality scores across the real and digital world. Since meaningful mean comparisons require at least scalar invariance, we estimated four MM‐LST‐RF models that differed only in their measurement invariance assumptions across fixed situations. Invariance was evaluated sequentially using Chen's (2007) criteria for large samples (*N* > 300) where metric non‐invariance is indicated by a ΔCFI of −0.010 or more, along with either a ΔRMSEA of 0.015 or more or a ΔSRMR of 0.030 or more; for scalar and residual invariance, the SRMR threshold lowers to 0.010. Model comparisons supported scalar invariance. The transition from time to metric invariance (ΔCFI = −0.002, ΔRMSEA = 0.000, ΔSRMR = 0.006) and from metric to scalar invariance (ΔCFI = −0.000, ΔRMSEA = 0.007, ΔSRMR = 0.001) were both within acceptable thresholds. However, the comparison of scalar with residual invariance exceeded the CFI threshold (ΔCFI = −0.012), indicating that residual invariance could not be assumed. Consequently, we based all further analyses on the MM‐LST‐RF model assuming scalar invariance across fixed situations.

The suitability of traditional model fit benchmarks for complex models has previously been critiqued (e.g., West et al. 2023). To address these concerns, several alternatives have been proposed. One approach involves calculating adjusted *χ* ^2^, RMSEA, and corresponding *p*‐values for models with many manifest variables (Yuan et al. 2015), which can be done in R using **lsttheory::correctedfit()**. Another option is to apply dynamic fit index cutoffs for CFI, RMSEA, and SRMR, available for certain model types via the **dynamic** package (Wolf and McNeish 2023). Beyond these, Millsap (2012) outlines a more general simulation‐based procedure to establish benchmarks for any fit index given any model and data type. In our case, the adjusted indices yielded the same conclusions as the traditional benchmarks. For simplicity, we therefore continue evaluating model fit using traditional benchmarks.

Following benchmarks commonly used by researchers across Europe, we defined acceptable fit as *χ* ^2^/df ≤ 3, CFI and TLI ≥ 0.90, RMSEA ≤ 0.08, and SRMR ≤ 0.10 (Gäde et al. 2020; Schweizer 2010). These benchmarks were met by our MM‐LST‐RF model (*χ* ^2^/df = 2.09, CFI = 0.94, TLI = 0.93, RMSEA = 0.05, SRMR = 0.09). With acceptable fit established, we turn to the model parameters shown further down in the *Model Fit & Parameters* panel. Since we established scalar invariance across fixed situations, we can compare mean levels of Negative Emotionality across offline and online contexts. On average, mean levels of Negative Emotionality are higher in the real world (*M_T1*/*M*(*T*_111_) = 3.64; output notation shown left of slash) than in the digital world (*M_T2*/*M*(*T*_112_) = 3.22). This difference is statistically significant (*z* = −10.19, *p* < 0.001) and corresponds to approximately half a scale point (*MDif_T2*/*M*(*T*_112_ –*T*_111_) = −0.43). The standardized beta coefficient additionally shows a significant, negative person‐by‐fixed situation interaction (*b1_T*/β_1112_ = −0.40), suggesting a medium‐sized buffering effect where individuals with higher offline Negative Emotionality tend to show smaller differences between contexts.

Next, we examine the model coefficients in the *Model Summary & Coefficients* panel (Figure 6A). In our example, 69% of trait‐like variance is shared across contexts (*Comm T1* × *T2*/*Comm*(*T*_112_) = 0.69), whereas 31% is fixed situation‐specific (*SitSpe T1* × *T2*/*SitSpe*(*T*_112_) = 0.31). The same panel reports indicator‐specific coefficients (Figure 6A displays those of the first six indicators). Item reliabilities range from 0.56 to 0.89 and are generally higher in the offline (*M*(*Rel*_*off*_) = 0.78) than in the online context (*M*(*Rel*_*on*_) = 0.68). Similarly, consistencies are higher for reference indicators offline (*M*(*Con*_*off*_) = 0.78) than for online (*M*(*Con*_*on*_) = 0.64), indicating that 78% and 64% of their variance, respectively, is attributable to the latent trait factor. Non‐reference indicators show much lower consistencies across both contexts (*ConY*/*Con*(*Y*_*imts*_) = 0.12–0.34), approximately 22%, highlighting a greater influence of method‐specific variance. Correspondingly, the estimated variances of the trait‐method factors are substantial (*V_TMims*/*Var*(*TM*_*ims*_) = 0.40–0.65; not shown in Figure 6). Finally, specificities are low across all indicators, with only about 4% of variance explained by occasion‐specific factors (*SpeY*/*Spe*(*Y*_*imts*_) = 0.01–0.11).

Given these results, we can begin interpreting the output to answer our research questions. In other scenarios, users may want to further refine their models or run comparisons between alternative specifications. In such cases, models can be estimated in the shiny app as usual, and the corresponding **lavaan** syntax can be copied from the *lavaan Syntax* panel (Figure 6C) into R for further customization. Alternatively, users can bypass the shiny app altogether by directly using the **mmLSTrf()** function in R. The generated syntax can be modified—for instance, by simplifying the model structure (e.g., omitting latent variables beyond occasion‐method factors) or adding covariates. The refined model can then be estimated using the **lavaan::sem()** function. If the models are nested (i.e., one can be derived from the other by adding constraints), the models can be formally compared using a *χ* ^2^ difference test by passing the models to the **anova()** function,^13^ in order from least to most restrictive. For non‐nested models, *χ* ^2^ difference testing is inappropriate, and model comparisons are typically based on information criteria such as AIC or BIC, which are available in the *Model Fit & Parameters* panel. Alternatively, non‐nested models can be compared using tests based on Vuong's (1989) theory, available in the **nonnest2** package in R (Merkle et al. 2016).

We emphasize that caution is necessary when making post hoc model modifications. Any modifications to the original model should be explicitly reported and, ideally, supported by theoretical reasoning. Tests of exploratory modifications must be clearly distinguished from confirmatory tests of the originally hypothesized model to maintain transparency and prevent bias resulting from sample‐specific relationships that may not replicate in other samples. Clearly documenting any adjustments to the original model helps avoid distorting results and keeps findings reliable and replicable. Preregistration of studies can further reduce unnecessary modifications by setting clear analysis plans in advance.

## 3 Discussion

### 3.1 Discussion of Results

In our motivational example, we examined trait‐level differences in Negative Emotionality across offline and online contexts, the generalizability of these differences while accounting for method effects, and potential person‐by‐fixed situation interactions. Scalar invariance across contexts was established, indicating that latent trait differences reflect true variation and allowing for meaningful trait‐level comparisons. As hypothesized in research question one, Negative Emotionality was lower online. This may reflect the possibility that digital environments afford more control over one's surroundings (Bayer et al. 2020; McFarland and Ployhart 2015), allowing individuals to act more consistently with their traits (Amichai‐Hamburger et al. 2002), thus reducing negative emotions.

Consistent with research question two, we found that these differences were *not* consistent across individuals; individuals higher in Negative Emotionality offline showed smaller trait‐level differences across contexts than individuals lower in Negative Emotionality offline. This finding contrasts with earlier research, which suggests more emotional relief in online settings among highly neurotic individuals (Blumer and Döring 2012; Rice and Markey 2009). However, those studies focused on computer‐mediated communication, whereas our study considered the broader digital context. It may be that individuals scoring high on Negative Emotionality benefit more from computer‐mediated interactions but that these benefits do not generalize to other domains of digital life. Additionally, the MM‐LST‐RF approach, unlike cross‐sectional, single‐method studies, may better isolate pure interaction effects, thus contributing to these discrepant findings.

For our third research question, we found that Negative Emotionality largely generalized across the real and digital world. Still, nearly one‐third of its variance was context‐specific. In line with prior findings (Deinzer et al. 1995; van Bavel et al. 2016), this highlights the context sensitivity of psychological constructs like the Big Five and underscores the need to consider fixed situations in personality research.

Beyond our primary research questions, we made several noteworthy observations, which were purely exploratory and should be put to the test in further replications. At the item level, reliabilities were slightly lower online, likely because the items were originally developed for offline contexts and may not be as well‐suited to capturing the construct in the digital world. Additionally, non‐reference indicators showed low consistencies despite similar specificities and reliabilities, indicating strong method effects. This result was supported for both true‐ and false‐keyed items. Finally, occasion‐method factor variances were near zero, indicating stable method effects over time, which resulted in their exclusion from the model. Findings on method effects are, of course, limited to the use of true‐ and false‐keyed items; different patterns may arise with other methods, such as self‐ versus other‐ratings.

### 3.2 General Discussion

Our analysis demonstrates the MM‐LST‐RF model's potential for studying person characteristics, method and occasion effects, as well as their interactions. To help overcome practical challenges hindering its implementation, we introduced a user‐friendly shiny app based on the new **mmLSTrf()** function from the **lsttheory** package. Our tutorial has several key strengths that help make the complex structure of MM‐LST‐RF models more accessible. We introduced multimethod and multi‐situation model implementations in a modular way, making it easier for readers to understand and apply these models step‐by‐step. The use of an empirical data example additionally helped illustrate key concepts of the approach, connecting methodological theory to substantial research questions regarding the digitalization of daily life. A detailed guide on the shiny app further simplified the implementation of MM‐LST‐RF models by enabling users to easily apply model restrictions, calculate key coefficients, and automate syntax generation, thus removing many of the barriers to the estimation of these models. The shiny app supports researchers in conducting more comprehensive studies and encourages thorough consideration of factors like methods and situations, ultimately promoting well‐founded and replicable results.

Overall, the MM‐LST‐RF model is a flexible tool for addressing diverse research questions beyond our example of Negative Emotionality in offline and online contexts. In its first empirical application, Hintz et al. (2019) used the model to study affect before and after smoking cessation, using differently keyed items across multiple occasions. While their study demonstrates its broad applicability, the MM‐LST‐RF model is a particularly valuable tool for advancing personality research. Researchers could, for example, measure agreeableness in politically charged versus neutral dyadic discussions using self‐, partner‐, and observer ratings from multiple measurement occasions to investigate whether agreeableness varies in contentious contexts and how these variations align or diverge across perspectives. Similarly, bicultural individuals' assertiveness could be examined in heritage versus mainstream cultural contexts via linguistic markers across communication platforms, shedding light on cultural influences on assertiveness and communication style. Another scenario would be participants reporting well‐being over several weeks during social and solitary leisure activities, using both positively and negatively worded items to account for method effects. Additionally, assessing extraversion would allow researchers to examine its moderating role on potential interactions between well‐being and leisure context. As a final example, self‐esteem could be assessed following success or failure feedback, using different scales administered repeatedly over extended intervals to help disentangle the effects of evaluative feedback on self‐esteem from temporary fluctuations and method‐related measurement artifacts.

Across these examples in personality research and potential applications in other areas of psychology, the MM‐LST‐RF model allows researchers to explore the stability and variability of psychological constructs over time, assess their generalizability across different fixed situations, examine the influence of measurement methods, and identify interactions between person, fixed situation, and method factors. This versatility highlights the potential of MM‐LST‐RF models to move beyond traditional interactionist approaches and incorporate the full scope of Ozer's (1986) simplified four‐dimensional data box to gain a more nuanced understanding of personality.

Future studies might examine the model's suitability for analyzing long‐term longitudinal data. In principle, researchers are free to select time intervals that align with their research objectives, theoretical considerations, and the nature of the measured construct, since MM‐LST‐RF models do not impose restrictions regarding the time periods between assessments. They do, however, rely on assumptions of time invariance, which often fail in studies of children across different developmental stages (e.g., Patterson 1993). Accordingly, evaluating the applicability of MM‐LST‐RF models in different stages over the lifespan would be an important next step. Moreover, as noted already by Hintz et al. (2019), a multitrait version of the MM‐LST‐RF model would offer additional functionality by also addressing issues of discriminant validity. However, given the added complexity, such models may be more challenging to specify, estimate, and interpret.

Another promising direction would be using a reference method approach (Courvoisier et al. 2008; Eid 2000; Eid et al. 2003) for MM‐LST‐RF models rather than reference indicators. This would have the advantage of reducing model complexity but would also alter the specification and interpretation of the method factors. Simulation studies would be valuable to assess the feasibility and effectiveness of this adjustment. If effective, the **mmLSTrf()** function could be extended to incorporate such MM(−1)‐LST‐RF models. Adding the option to estimate LST‐RF or MM‐LST models individually may also be a possible future extension. This was not included in the current version, as the focus was primarily on enhancing the accessibility of the more complex MM‐LST‐RF model. However, providing this functionality could, in the future, offer greater flexibility when working on research questions that require different model specifications.

### 3.3 Conclusion

MM‐LST‐RF models offer researchers a powerful tool for analyzing data across persons, (fixed) situations, methods, and time. Our tutorial provides foundational knowledge and practical guidance for applying these models, supported by software tools that reduce entry barriers. We hope this work advances longitudinal multimethod and multi‐situation research, promoting a more thorough understanding of psychological constructs.

## Author Contributions

**Dora L. Tinhof:** conceptualization, data curation, formal analysis, investigation, methodology, software, visualization, writing – original draft, writing – review and editing. **Axel Mayer:** conceptualization, software, supervision, writing – review and editing.

## Ethics Statement

The function code as well as both example datasets are part of the lsttheory package available under https://github.com/amayer2010/lsttheory. The study from which the empirical data example was drawn was approved by the Bielefeld University Ethics Committee (reference number 2023‐032).

## Conflicts of Interest

The authors declare no conflicts of interest.

## Supporting information


Data S1.

## Endnotes

^1^
The preregistration can be found on the Open Science Framework (https://doi.org/10.17605/OSF.IO/XKCAV).

^2^
For simplicity, the other‐ratings are not considered in this motivational example.

^3^
Readers may consult Enders (2022) for more information on dealing with missing data involving special missingness mechanisms.

^4^
For a discussion of strategies on handling attrition in longitudinal designs see Graham (2009).

^5^
This applies to single‐trait models. For multitrait models, refer to Geiser et al. (2015).

^6^
We use the deviation term to refer to a deviation from the predicted trait level. It does not imply a difference score.

^7^
Contrary to the deviation interpretation of occasion, trait‐method and occasion‐method factors, this variable can be interpreted as difference score.

^8^
Nonlinear interaction effects may still be present even when β_12_ = 0 (Geiser et al. 2015).

^9^
MM‐LST models can also be applied to multiple constructs, enabling the study of convergent and discriminant validity (Courvoisier et al. 2008). However, as this tutorial focuses on single‐construct MM‐LST‐RF models, we only present the single‐construct version of MM‐LST models here.

^10^
Unlike in classic LST models, the consistency and specificity coefficients do not sum up to the reliability value since method variance is excluded from the calculation of consistency and specificity, but is included in the calculation of reliability.

^11^
Trait and trait‐method factor covariances will not be estimated when interaction effects have been requested for them, even if selected. However, model‐implied covariances are still derived from the model parameters.

^12^
Assuming essential equivalence across latent variables automatically implies metric invariance—as loadings remain consistent across occasions, methods, and conditions. Setting trait factors to be equivalent implies scalar invariance, and treating them as parallel implies residual invariance.

^13^
When comparing models estimated with the **mmLSTrf()** function, they need to be passed to the **anova()** function in the following format: **model@lavaanres**. This may, for example, be the case when comparing nested MM‐LST‐RF models with different equivalence assumptions.

## Appendix A

**TABLE A1:** Selected model coefficients of MM‐LST‐RF models.

Coefficient	Equation	Description
*Var*(*Y*_*imts*_)^a^	$\lambda_{\mathrm{ims}}^{2}\;\mathrm{Var}\left(T_{11s}\right)+\delta_{\mathrm{ims}}^{2}\;\mathrm{Var}\left(O_{11s}\right)+\mathrm{Var}\left({\mathrm{TM}}_{\mathrm{ims}}\right)+$ $\gamma_{\mathrm{ims}}^{2}\mathrm{Var}\left({\mathrm{OM}}_{\mathrm{mts}}\right)+\varepsilon_{\text{imts}}$	**Variance:** Total variance of manifest variable *Y*_*imts*_
*Con*(*Y*_*imts*_)	$\frac{\lambda_{\mathrm{ims}}^{2}\;\mathrm{Var}\left(T_{11s}\right)}{\mathrm{Var}\left(Y_{\text{imts}}\right)}$	**Consistency:** Proportion of stable variance explained by *T*_*11s*_
*Spe*(*Y*_*imts*_)	$\frac{\delta_{\mathrm{ims}}^{2}\;\mathrm{Var}\left(O_{11s}\right)}{\mathrm{Var}\left(Y_{\text{imts}}\right)}$	**Specificity:** Proportion of variable variance explained by *O*_*11ts*_
*Rel*(*Y*_*imts*_)^a^	$\frac{\lambda_{\mathrm{ims}}^{2}\;\mathrm{Var}\left(T_{11s}\right)+\delta_{\mathrm{ims}}^{2}\;\mathrm{Var}\left(O_{11s}\right)+\mathrm{Var}\left({\mathrm{TM}}_{\mathrm{ims}}\right)+\gamma_{\mathrm{ims}}^{2}\mathrm{Var}\left({\mathrm{OM}}_{\mathrm{mts}}\right)}{\mathrm{Var}\left(Y_{\text{imts}}\right)}$	**Reliability:** Proportion of total explainable variance
*Comm*(*T*_*11s*_)	[*Corr*(*T*_*111*_, *T*_*11s*_)]^2^	**Commonality:** Proportion of trait‐like variance, which is common across fixed situations
*SitSpe*(*T*_*11s*_)	1 − [*Corr*(*T*_*111*_, *T*_*11s*_)]^2^	**Fixed Situation Specificity:** Proportion of trait‐like variance, which is fixed situation‐specific
*Comm*(*TM*_*ims*_)	[*Corr*(*TM*_*im1*_, *TM*_*ims*_)]^2^	**Method Commonality:** Proportion of variance in trait‐method effects which is shared across fixed situations
*SitSpe*(*TM*_*ims*_)	1 − [*Corr*(*TM*_*im1*_, *TM*_*ims*_)]^2^	**Method Fixed Situation Specificity:** Proportion of variance in trait‐method effects which is fixed situation‐specific

^a^ Reference indicators do not load onto *TM*_*ims*_ or *OM*_*ims*_. Additionally, their loadings λ_ims_ and δ_ims_ are fixed to 1.
