# Supplementary material for: Making Multimethod Latent State–Trait Models for Random and Fixed Situations Accessible: A Tutorial
Source: J Pers. 2025 Jun 16;93(5):1018–41. doi: 10.1111/jopy.13031 (PMC12421706; doi:10.1111/jopy.13031)
Supplement: Supplementary file 1 — Data S1. [file JOPY-93-1018-s001.pdf]

Making multi-method latent state-trait models for fixed and random situations accessible: a tutorial

Supplement

Dora L. Tinhof[1] and Axel Mayer[1]

[1]Department of Psychological Methods and Evaluation, Faculty of Psychology and Sport Science, Bielefeld University

Author Note

Dora L. Tinhof https://orcid.org/0000-0001-8970-1863

Axel Mayer https://orcid.org/0000-0001-9716-878X

The function code as well as both example datasets are part of the lsttheory package available under https://github.com/amayer2010/lsttheory. The study from which the empirical data example was drawn was approved by the Bielefeld University Ethics Committee (reference number 2023-032).

The author(s) received no financial support for the research, authorship, and/or publication of this article. We declare no conflicts of interest. This research was not preregistered.

Correspondence concerning this article should be addressed to Dora Leander Tinhof, Department of Psychological Methods and Evaluation, Bielefeld University, Universitaetsstrasse 25, 33615 Bielefeld, Germany. Email: dora.tinhof@uni-bielefeld.de

The `mmLSTrf()` Function

This manual provides information on the **`mmLSTrf()`** function from the **`lsttheory`** package (Mayer, 2024). The package can be installed from GitHub using the following syntax.

```
install.packages("devtools")
devtools::install_github("amayer2010/lsttheory")
library(lsttheory)
```

The code below shows the function with all its arguments set to their default values. A summary of the arguments, input types, and default settings can be found in Table S1. Following, the arguments of the function are detailed.

```
mmLSTrf(data, nSit = 2, nTime = 2, nMth = 2, structural = "TF",
        includeOMF = TRUE, lat.cov = list(TFcov = FALSE, OFcov = FALSE,
        TMFcov = FALSE, OMFcov = FALSE), meanstructure = FALSE,
        meas.invar = "time.invar”, equiv.ass = list(TF = "par",
        OF = "ess.equiv", OMF = "ess.equiv"), addsyntax = "", ...)
```

data. The **`data`** argument requires a data frame in wide format, containing only the indicator variables relevant to the MM-LST-RF model. The variable order must follow the top-to-bottom order of indicators in a typical MM-LST-RF path diagram, where indicators are first grouped by fixed situations, then occasions, and then methods. The first indicator Y_{11ts} $(i = 1)$ of the first method $(m = 1)$, and the trait factor T_{111} of the first fixed situation $(s = 1)$ automatically serve as reference points. For further guidance on the required data structure, users may also refer to the function documentation, which is accessible via **`?mmLSTrf()`**.

The **`lsttheory`** package includes two example datasets for the **`mmLSTrf()`** function. The first, **`mmLSTrf_RealDataExample`** ($N = 425$), comprises 24 variables from an empirical study measuring Negative Emotionality at two occasions across both the real (offline) and digital (online) world, using three true- and three false-keyed items. The second dataset, **`mmLSTrf_SimulatedDataExample`** ($N = 500$), is a simulated dataset with 36 variables generated based on the parameter values shown in Table S2. Additional details on either dataset can be accessed via **`?mmLSTrf_RealDataExample`** or **`?mmLSTrf_SimulatedDataExample`**, respectively.

`nSit, nMth, nTime.` The arguments **`nSit`**, **`nMth`**, and **`nTime`** specify the number of fixed situations, methods, and occasions; at least two of each are required for model estimation. The function assumes a balanced design with an equal number of indicators across methods, an equal number of methods across occasions, and an equal number of occasions across fixed situations.

Table S1. *Arguments of the* `mmLSTrf()` *function*

Argument	Use	Input Type	Options	Default
**`data`**	dataset containing observed variables	data frame	-	-
**`nSit`**	number of fixed situations	integer	nSit ≥ 2	2
**`nTime`**	number of occasions	integer	nTime ≥ 2	2
**`nMth`**	number of methods	integer	nMth ≥ 2	2
**`structural`**	estimation of interaction effects	character string	`"none"` || `"TF"` || `"TMF"` || `"both"`	`"TF"`
**`includeOMF`**	inclusion of occasion-method factors	logical	`TRUE` || `FALSE`	`TRUE`
**`lat.cov`**	estimation of covariances between latent variables	list	`TFcov = TRUE` || `FALSE`, `OFcov = TRUE` || `FALSE`, `TMFcov = TRUE` || `FALSE`, `OMFcov = TRUE` || `FALSE`	`FALSE`
**`meanstructure`**	estimation of meanstructure	logical	`TRUE` || `FALSE`	`FALSE`
**`meas.invar`**	measurement invariance restrictions	character string	`"time.invar"` || `"metric.m"` || `"metric.s"` || `"metric.b"` || `"scalar.m"` || `"scalar.s"` || `"scalar.b"` || `"residual.m"` || `"residual.s"` || `"residual.b"`	`time.invar`
**`equiv.ass`**	equivalence assumptions	list	`TF = "cong"` || `"ess.equiv"` || `"equiv"` || `"ess.par"` || `"par"`, `OF = "cong"` || `"ess.equiv"`, `OMF = "cong"` || `"ess.equiv"`[a]	`TF = "par"`; `OF & OMF = "ess.equiv"`
**`addsyntax`**	custom model syntax	character string	-	`""`
**`(...)`**	further arguments to `lavaan::sem()`	`lavaan` function arguments	e.g., `estimator = "MLR"`, `missing = "ML"`, `se = "robust"`	-

Note. [a] Trait-method factors are not listed since their loadings are always restricted to 1 due to time-invariance assumptions coupled with identification constraints. *TF* = trait factor, *OF* = occasion factor, *OMF* = occasion-method factor.

`structural`. The structural argument specifies whether person-by-fixed situation or fixed situation-by-method interaction effects are estimated. Setting structural to **`"TF"`** estimates person-by-fixed situation interactions, with the first fixed situation serving as comparison standard. Setting it to **`"TMF"`** estimates method-by-fixed situation interactions, where trait-method factors from the first fixed situation serve as reference point. For instance, TM_{312} would be compared to TM_{311}. If both

types of interaction are of interest, the option **"both"** can be selected. The model can also be estimated without latent difference variables by setting structural to **"none"**. In this case, latent variable correlations can be specified using **lat.cov** or **addsyntax**.

Table S2. *Parameter values of the simulated data example*

Parameter	Value	Parameter	Value
$E(T_{111})$	2.75	$Comm(T_{112})$	0.44
$E(T_{112})$	3.25	$\varepsilon_{\text{imts}}$	0.15
$Var(T_{111})$	0.40	α_{ims}	0.00
$Var(T_{112})$	0.45	λ_{ims}	1.00
$Var(O_{11t1})$	0.20	δ_{ims}	1.00
$Var(O_{11t2})$	0.30	γ_{ims}	1.00
$Var(TM_{ims})$	0.10	β_{1112}	0.35
$Var(OM_{mts})$	0.10	β_{0112}	1.31
$Var(\omega_{112})$	0.25		

Note. Simulation seed = 548263; $N = 500$; Trait factors T_{11s} are simulated to be essentially parallel, all other latent variables are essentially equivalent. Scalar measurement invariance holds across fixed situations & methods. Latent variables are orthogonal apart from trait factors T_{11s}. i = indicator, m = method, t = occasion, s = fixed situation.

includeOMF. The **includeOMF** argument controls if occasion-method factors are estimated; by default, they are included in the model. However, to improve model parsimony researchers may choose to exclude them based on theoretical considerations or if previous estimation suggests that their variances are negligible. Excluding occasion-method factors reflects the assumption that there are no relevant occasion-specific method effects.

lat.cov. The **lat.cov** argument allows estimation of covariances between latent variables for each of the four latent variable types. By default, all latent variables are orthogonal. If set to **TRUE**, all admissible covariances of the specified type will be estimated. Trait factors and trait-method factors will correlate with all other factors of the same type, while occasion and occasion-method factors will correlate with their counterparts in other fixed situations. Note that **TFcov** and **TMFcov** are automatically set to **FALSE** when **structural = "TF"** or **"both"**, and when **structural = "TMF"** or **"both"**, respectively – even if manually set to **TRUE** – since regression parameters β_{111s} are estimated instead. Model-implied covariances are still derived from the model parameters.

It is advisable to use this option sparingly, particularly for trait-method factors, as estimating too many covariances substantially increases the number of free parameters and potentially impairs model performance. To estimate only a selected number of covariances or covariances between trait factors and trait-method factors across different fixed situations, custom syntax can be passed to the function via the **addsyntax** argument.

meas.invar. The **meas.invar** argument specifies different levels of measurement invariance

assumptions. Four increasingly restrictive levels of measurement invariance – time invariance, metric/weak factorial invariance, scalar/strong factorial invariance, residual/strict invariance – can be selected. Besides time invariance – the function default – each of the remaining levels can either be applied across methods, fixed situations, or both. Table S3 provides an overview of all available options, while formal equations describing their implications can be found in Table S4. Partial measurement invariance can also be implemented by specifying a less restrictive measurement invariance level and adding custom restrictions via the `addsyntax` argument.

Table S3. *Specification options for the measurement invariance argument* `meas.invar`

Level	Argument[a]	Assumption
Time	`time.invar`	Assumes time invariance of indicator loadings & intercepts across occasions.[b]
Metric	`metric.m`	Assumes time invariance plus equal loadings across methods within each fixed situation.
	`metric.s`	Assumes time invariance plus equal loadings across fixed situations.
	`metric.b`	Assumes time invariance plus equal loadings across methods & fixed situations.
Scalar	`scalar.m`	Assumes time invariance plus equal loadings & intercepts across methods within each fixed situation.
	`scalar.s`	Assumes time invariance plus equal loadings & intercepts across fixed situations.
	`scalar.b`	Assumes time invariance plus equal loadings & intercepts across methods & fixed situations.
Residual	`residual.m`	Assumes time invariance plus equal loadings, intercepts & error variances across methods within each fixed situation.
	`residual.s`	Assumes time invariance plus equal loadings, intercepts & error variances across fixed situations.
	`residual.b`	Assumes time invariance plus equal loadings, intercepts & error variances across methods & fixed situations.

Note. [a] `.m` = assumptions made only across methods; `.s` = assumptions made only across fixed situations; `.b` = assumptions made across methods & fixed situations. [b] There is no time invariance assumption for ε_{imts}.

Table S4. *Implications of measurement invariance assumptions for model parameters*

Assumption[a]	Loadings			Intercepts	Errors
`metric.m`	$\lambda_{ims} = \lambda_{is}$	$\delta_{ims} = \delta_{is}$	$\gamma_{ims} = \gamma_{is}$	-	-
`metric.s`	$\lambda_{ims} = \lambda_{im}$	$\delta_{ims} = \delta_{im}$	$\gamma_{ims} = \gamma_{im}$	-	-
`metric.b`	$\lambda_{ims} = \lambda_{i}$	$\delta_{ims} = \delta_{i}$	$\gamma_{ims} = \gamma_{i}$	-	-
`scalar.m`	$\lambda_{ims} = \lambda_{is}$	$\delta_{ims} = \delta_{is}$	$\gamma_{ims} = \gamma_{is}$	$\alpha_{ims} = \alpha_{is}$	-
`scalar.s`	$\lambda_{ims} = \lambda_{im}$	$\delta_{ims} = \delta_{im}$	$\gamma_{ims} = \gamma_{im}$	$\alpha_{ims} = \alpha_{im}$	-
`scalar.b`	$\lambda_{ims} = \lambda_{i}$	$\delta_{ims} = \delta_{i}$	$\gamma_{ims} = \gamma_{i}$	$\alpha_{ims} = \alpha_{i}$	-
`residual.m`	$\lambda_{ims} = \lambda_{is}$	$\delta_{ims} = \delta_{is}$	$\gamma_{ims} = \gamma_{is}$	$\alpha_{ims} = \alpha_{is}$	$\varepsilon_{imts} = \varepsilon_{its}$
`residual.s`	$\lambda_{ims} = \lambda_{im}$	$\delta_{ims} = \delta_{im}$	$\gamma_{ims} = \gamma_{im}$	$\alpha_{ims} = \alpha_{im}$	$\varepsilon_{imts} = \varepsilon_{imt}$
`residual.b`	$\lambda_{ims} = \lambda_{i}$	$\delta_{ims} = \delta_{i}$	$\gamma_{ims} = \gamma_{i}$	$\alpha_{ims} = \alpha_{i}$	$\varepsilon_{imts} = \varepsilon_{it}$

Note. [a] Time invariance is not listed since loadings and intercepts are time invariant per default in MM-LST-RF models and therefore do not have a subscript *t*. Measurement invariance can be fixed across methods (`.m`), across fixed situations (`.s`) or across both (`.b`).

equiv.ass. The **equiv.ass** argument allows for the specification of equivalence assumptions. Same as **meas.invar**, **equiv.ass** imposes constraints on loadings, intercepts, and error variances. However, while **meas.invar** fixes parameters across conditions (methods or fixed situations), **equiv.ass** restricts parameters of indicators measuring the same latent factor (trait, occasion, or occasion-method factors) to uniformity. Five increasingly restrictive levels of equivalence assumptions - congenericity, essential equivalence, equivalence, essential parallelity, and parallelity - can be selected. They are summarized in Table S5 and formalized in Table S6.

Table S5. *Specification options for the equivalence assumptions argument* `equiv.ass`

Latent Variable[a]	Argument	Assumption
TF, OF, OMF	**cong**	*Congeneric* measures only make identification restrictions, fixing the loading of the first indicator of the latent variable to 1 and the intercept to 0 for TF.
	**ess.equiv**	*Essentially equivalent* measures assume equal loadings of all indicators of the latent variable.
TF	**equiv**	*Equivalent* measures assume equal loadings & intercepts of all indicators of TF.
TF	**ess.par**	*Essentially parallel* measures assume equal loadings & error variances of all indicators of TF.
TF	**par**	*Parallel* measures assume equal loadings, intercepts & error variances of all indicators of TF.

Note. [a] Latent variable type which the assumption can be applied to. *TF* = trait factor, *OF* = occasion factor, *OMF* = occasion-method factor.

Table S6. *Implications of equivalence assumptions for model parameters*

Assumption[a]	Latent Variable[b]	Loadings	Intercepts	Errors
**ess.equiv**	TF	$\lambda_{ims} = \lambda_s = 1$	-	-
	OF	$\delta_{ims} = \delta_s = 1$	-	-
	OMF	$\gamma_{ims} = \gamma_{ms} = 1$	-	-
**equiv**	TF	$\lambda_{ims} = \lambda_s = 1$	$\alpha_{ims} = \alpha_s = 0$	-
**ess.par**	TF	$\lambda_{ims} = \lambda_s = 1$	-	$\varepsilon_{imts} = \varepsilon_s$
**par**	TF	$\lambda_{ims} = \lambda_s = 1$	$\alpha_{ims} = \alpha_s = 0$	$\varepsilon_{imts} = \varepsilon_s$

Note. [a] Congenericity (“`cong`”) is not listed since it is the standard assumption in MM-LST-RF models. [b] Loadings of trait-method factors are not listed because they are always fixed to 1. Due to identification restrictions, the equivalence assumptions result in all loadings = 1 & intercepts = 0 when targeted by the applied restrictions. “`ess.equiv`” = essential equivalence, “`equiv`” = equivalence, “`ess.par`” = essential parallelity, “`par`” = parallelity; *TF* = trait factor, *OF* = occasion factor, *OMF* = occasion-method factor.

The assumptions **cong** and **ess.equiv** affect only loadings and can thus be applied to trait factors, occasion factors, and occasion-method factors. The remaining assumptions also constrain intercepts and/or error variances and apply only to trait factors since occasion factors and occasion-method factors are defined as residuals of the trait factors with means of zero. As all trait-method factor loadings are automatically fixed to one – for identification purposes and due to time invariance

assumptions – no further restrictions can be applied to them.

The most restrictive assumptions are set as function default values (i.e., **`TF = "par"`**, **`OF`** & **`OMF = "ess.equiv"`**). Should the initial model perform poorly, restrictions can be gradually relaxed and model fit can be compared using the **`anova()`** function like shown below. Entered models need to be ordered from least to most restrictive.

```
anova(mod1@lavaanres, mod2@lavaanres, mod3@lavaanres)
```

`meanstructure`. Setting **`meanstructure`** to **`TRUE`** estimates a mean structure for the model. This is necessary for mean comparisons. Restrictions on intercept parameters only apply when the mean structure is included, as intercepts are absent from the model otherwise.

`addsyntax`. The **`addsyntax`** argument allows for the manual input of custom **`lavaan`** syntax which overwrites conflicting specifications stemming from the other arguments. The used function syntax notation of model parameters necessary for custom input is summarized in Table S7 and can also be found in the function documentation accessible via **`?mmLSTrf`**.

`...` Standard arguments from the **`lavaan`** package (Rosseel, 2012) may be passed to the function using this argument, like specifying estimators (**`estimator = “MLR”`**), missing data mechanisms (**`missing = “ML”`**), robust standard errors (**`se = “robust”`**), etc.

Output. Printing the estimated model using **`print(model)`** provides a brief model summary as well as the MM-LST-RF model coefficients. For a full summary, including fit indices and (standardized) parameter estimates, **`summary(model@lavaanres`**, **`fit.measures = TRUE`**, **`standardized = TRUE)`** can be used. Additionally, the automatically generated **`lavaan`** syntax is accessible via **`cat(model@lavaansyntax)`**. It can be used as blueprint to extend the model beyond the possibilities of the function and estimate the adapted syntax using **`lavaan::sem()`**.

Table S7. *Syntax notation of model parameters in* `mmLSTrf()`

Parameter	Model Notation	Syntax Notation	Example
Observed variable	Y_{imts}	Y_imts	given variable-name[a]
Trait factor	T_{11s}	Ts	T2
Occasion factor	O_{11ts}	Ots	O32
Trait-method factor	TM_{ims}	TMims	TM212
Occasion-method factor	OM_{mts}	OMmts	OM132
Trait factor mean	$Mean(T_{11s})$	M_Ts	M_T2
Trait factor variance	$Var(T_{11s})$	V_Ts	V_T2
Occasion factor variance	$Var(O_{11ts})$	V_Ots	V_O32
Trait-method factor variance	$Var(TM_{ims})$	V_TMims	V_TM212
Occasion-method factor variance	$Var(OM_{mts})$	V_OMmts	V_OM132
Observed variable variance	$Var(Y_{imts})$	V_Y_imts	V_(variable-name)[a]
Trait factor covariance	$Cov(T_{11s}, T_{11s})^b$	Cv_TaxTb	Cv_T2xT3
Occasion factor covariance	$Cov(O_{11ts}, O_{11ts})^b$	Cv_OaxOb	Cv_O32xO33
Trait-method factor covariance	$Cov(TM_{ims}, TM_{ims})^b$	Cv_TMaxTMb	Cv_TM212xTM213
Occasion-method factor covariance	$Cov(OM_{mts}, OM_{mts})^b$	Cv_OMaxOMb	Cv_OM132xOM133
Residual variance epsilon	ε_{imts}	eps_imts	eps_2132
Intercept alpha	α_{ims}	alph_ims	alph_212
Loading lambda	λ_{ims}	lam_ims	lam_212
Loading delta	δ_{ims}	del_ims	del_212
Loading gamma	γ_{ims}	gam_ims	gam_212
**Trait factor change score parameters**			
Trait factor difference	$(T_{11s} - T_{111})$	Dif_Ts	Dif_T2[c]
Regression intercept alpha	β_{011s}	b0_Ts	b0_T2[c]
Regression slope beta	β_{111s}	b1_Ts	b1_T2[c]
Residual variance omega	$Var(\omega_{11s})$	omg_Ts	omg_T2[c]
**Trait factor change score parameters**			
Trait-method factor difference	$(TM_{ims} - TM_{im1})$	Dif_TMims	Dif_TM212[c]
Regression slope beta	β_{1ims}	b1_TMims	b1_T212[c]
Residual variance omega	$Var(\omega_{ims})$	omg_TMims	omg_T212[c]
**Coefficients**			
Commonality	$Com(T_{11s})^d$	Com_Ts	Com_T2[c]
Fixed situation specificity	$SitSpe(T_{11s})^d$	SitSp_Ts	SitSp_T2[c]
Method commonality	$Com(TM_{ims})^d$	Com_TMims	Com_TM212[c]
Method fixed situation specificity	$SitSpe(TM_{ims})^d$	SitSp_TMims	SitSp_TM212[c]
Observed variable reliability	$Rel(Y_{imts})$	RelY_imts	Rel(variable-name)[a]
Observed variable consistency	$Con(Y_{imts})$	ConY_imts	Con(variable-name)[a]
Observed variable specificity	$Spe(Y_{imts})$	SpeY_imts	Spe(variable-name)[a]

Note. [a] Variable names in the dataset are carried over to the model parameters associated with the observed variables. [b] Covariances are calculated between two distinct latent variables of the same variable type (denoted by a & b in the Syntax Notation column). [c] Only the latent variable of the non-reference situation appears in the parameter notation, as the reference situation is always implicitly included. [d] For commonality & fixed situation specificity, the fixed reference situation is always $s = 1$.
